# Atomic structure of human TOM core complex

**DOI:** 10.1038/s41421-020-00198-2

**Published:** 2020-09-29

**Authors:** Wenhe Wang, Xudong Chen, Laixing Zhang, Jingbo Yi, Qingxi Ma, Jian Yin, Wei Zhuo, Jinke Gu, Maojun Yang

**Affiliations:** 1 Ministry of Education Key Laboratory of Protein Science, Beijing Advanced Innovation Center for Structural Biology & Frontier Research Center for Biological Structure, Tsinghua-Peking Joint Center for Life Sciences, School of Life Sciences, Tsinghua University, 100084 Beijing, China; 2 School of Pharmacy, Tongji Medical College, Huazhong University of Science and Technology, Wuhan, 430030 Hubei China

**Keywords:** Structural biology, Cryoelectron microscopy

## Abstract

The translocase of the outer mitochondrial membrane (TOM) complex is the main entry gate for mitochondrial precursor proteins synthesized on cytosolic ribosomes. Here we report the single-particle cryo-electron microscopy (cryo-EM) structure of the dimeric human TOM core complex (TOM-CC). Two Tom40 β-barrel proteins, connected by two Tom22 receptor subunits and one phospholipid, form the protein-conducting channels. The small Tom proteins Tom5, Tom6, and Tom7 surround the channel and have notable configurations. The distinct electrostatic features of the complex, including the pronounced negative interior and the positive regions at the periphery and center of the dimer on the intermembrane space (IMS) side, provide insight into the preprotein translocation mechanism. Further, two dimeric TOM complexes may associate to form tetramer in the shape of a parallelogram, offering a potential explanation into the unusual structural features of Tom subunits and a new perspective of viewing the import of mitochondrial proteins.

## Introduction

The translocase of the outer mitochondrial membrane (TOM) complex mediates the import of nuclear-encoded proteins into mitochondria. According to proteomic studies, mitochondria contain ~1000 (yeast) to 1500 (human) different proteins, of which 99% are encoded by nuclear genes and need to be imported after synthesis on cytosolic ribosomes^1–3^. TOM complex recognizes mitochondrial-targeted precursor proteins, mediates their entry, and transfers them to distinct protein translocation systems on the outer and inner mitochondrial membrane, including the sorting and assembly machinery (SAM complex) and the translocases of the inner membrane (TIM complex)^4–6^.

Unlike most membrane protein complexes, the TOM complex contains both α-helical and β-barrel integral membrane proteins^7,8^. Its β-barrel protein Tom40 forms the protein-conducting channel through which preproteins enter^9–11^. Its six other subunits are transmembrane α-helices: three receptor proteins (Tom20, Tom70, Tom22) that are involved in preprotein recognition, and three small Tom proteins (Tom5, Tom6, Tom7) that help with complex stability and assembly^1,5,12,13^. Recently, three fungal TOM core complex (TOM-CC) structures have been reported at medium to high resolutions^14–16^. The structures share the underlying architecture of a symmetrical dimer with two Tom40 pores, each surrounded by the transmembrane segments of Tom5, Tom6, and Tom7, and two Tom22 receptors connecting them at the dimer interface. It is worth noting, however, that the groups have reported varying oligomerization states.

Functional and structural studies in recent years have indicated that the mitochondrial protein import machinery is subject to detailed regulation and may play a regulatory role in metabolism, stress response, and pathogenesis of diseases^17,18^. In addition to being the channel-forming subunit of the TOM complex, Tom40 has been suggested to function independently in specific translocation pathways and is linked to multiple neurodegenerative diseases^19–21^. Tom22, on the other hand, has been shown to have interaction with the SAM complex and stimulates the formation of β-barrel proteins^22,23^. The *tomm22* gene has also been reported to be essential for hepatocyte survival and provides a model for liver regeneration^24^. Besides, Tom6 and Tom22 have been indicated to have direct contact, suggesting that aside from stabilizing the interaction of Tom40 with Tom22, Tom6 might have a more critical role in the assembly and maintenance of the TOM complex^25,26^. Other studies have indicated that Tom7 plays an antagonistic role against Tom6 in the assembly of TOM: whereas Tom6 promotes its formation, Tom7 delays it^6,26^. The exact mechanism through which Tom7 functions is unclear; however, its involvement in impairing the association of Mdm10 with SAM complex might be a possible explanation^27,28^.

Despite recent advances in examining the molecular mechanism of mitochondrial protein translocation^14–16^, the structure of the human TOM complex and the functions of each of its subunits remain to be elucidated. The structural information available to date regarding the complex mostly comes from structures resolved in fungi, which leaves room for further investigation of mitochondrial protein import in mammals. Here we report the cryo-EM structure of human TOM-CC at 3.4 Å resolution. In addition to the dimeric form of the complex, we observed a higher tetrameric oligomerization state at 8.5 Å resolution (Supplementary Figs. S1, 2 and Table S1), resembling that of the recently reported tetrameric structure in yeast^15^. Notably, through combining crosslinked mass spectrometry (CL-MS) results with structural evidence (Supplementary Table S2), we propose possible protein translocation pathways that contribute to a more comprehensive understanding of the human mitochondrial protein import mechanism.

### Overall structure of human TOM-CC

**Fig. 1:**
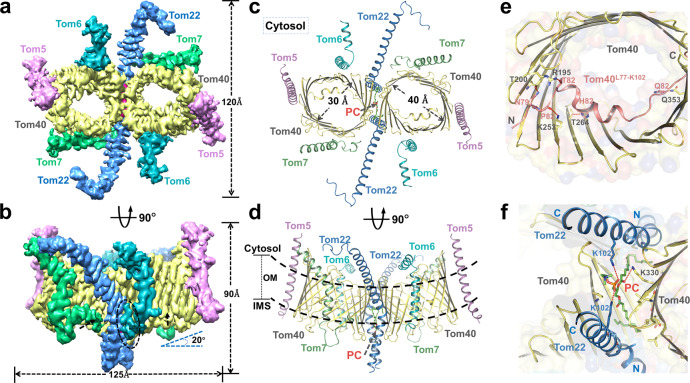
Overall structure of human Tom-CC. **a**, **b** A 3.4-Å-resolution cryo-EM reconstruction of the dimeric TOM complex. Tom subunits and measurements of TOM complex are indicated. A potential short peptide is indicated by dotted cycles in **b**. Shown are views from the cytosol. **c**, **d** Atomic model of the TOM complex in cartoon representation. A PC molecule between the Tom40 subunits is represented in sticks. The size of Tom40 pore is indicated in **c**. Dotted lines in **d**, approximate outer membrane (OM) boundaries. **e** Interactions between Tom40^L77–K102^ (color in salmon) and β-strand domains of Tom40. The polar interactions are indicated by red dotted lines. IMS views are shown. **f** Interactions between PC molecule and TOM complex. The polar interactions are indicated by red dotted lines. IMS views are shown.

Similar to previously reported cryo-EM structure of TOM-CC in fungi^14–16^, the human TOM-CC we solved at 3.4 Å resolution also takes the form of a centrosymmetric dimer, consisted of subunits Tom5, Tom6, Tom7, Tom22, and Tom40 (Fig. 1a, b, Supplementary Fig. S1b and Table S3). The complex has an overall dimension of ~125 Å × 120 Å × 90 Å and an approximate molecular weight of 150 kDa. The two Tom40 β-barrel proteins, embedded in the outer membrane at a 20° upward angle, form the main structure of the complex. The two Tom22 receptor subunits, located along the dimer interface, connect the Tom40 pores. Small protein subunits Tom5, Tom6, and Tom7 surround the outer wall of each Tom40, with Tom5 being at the distal end of the dimer, and Tom6 and Tom7 situating on opposite sides across the β-barrel channel (Fig. 1a–d).

The α-helical proteins of the complex possess distinct features. The transmembrane segment of Tom5 inserts into the outer membrane at a tilted angle following the inclined contour of Tom40, with its C-terminal end extending slightly away into the IMS (Fig. 1a–d, Supplementary Fig. S3a). Tom6, in addition to the transmembrane helix, has a cytosolic α-helical domain connected by a nine-residue loop (Tom6^F34–R42^) that runs parallel to the outer membrane (OM) of mitochondria, which is unobserved in its fungal counterpart^15,16^ (Fig. 1a–d, Supplementary Fig. S3b). Tom7 is of an interesting configuration, with a main α-helical segment connected to a short C-terminal α-helix by a fourteen-residue loop (Tom7^F36–V49^), which extends into the IMS. Both of Tom7 and Tom22 have notable kinks in their helical TM segments (Fig. 1a–d, Supplementary Fig. S3c, d), whose formation and biological function deserve careful analysis. Interestingly, looking down at the structure from the cytosolic side, we notice an overall trend in the orientation of the extended helices of the subunits, which together appear to sway in a clockwise direction (Fig. 1a, c). The pattern may have a broader significance in facilitating preprotein translocation, especially in the context of the higher tetrameric form.

Despite the low sequence homology between human and fungal TOM-CC (Supplementary Fig. S4), our structure demonstrates a high degree of structural homology with the recently reported fungal structures. The core complex is universally composed of the five subunits, with the dimeric form as a basic functional unit. However, the human and fungal TOM-CC have notable structural and conformational differences in their respective subunits, indicating potentially divergent preprotein translocation mechanism, which would be discussed below in detail.

### Dimeric Tom40 pores

The two elliptical pores at the center of the complex are formed by β-barrel protein Tom40, each with an inner cross-sectional diameter of 40 Å by 30 Å, excluding the N-terminal α-helix (Fig. 1c). The β-barrel protein consists of 19 transmembrane β-strands, characteristic of the voltage-dependent anion channel (VDAC)-porin superfamily of β-barrel proteins^29^. At the N-terminus preceding the first β strand, an α-helical segment extends through the β-barrel pore into the IMS (Fig. 1e). In comparison to the two recently reported yeast Tom40 structures^15,16^, our structure is relatively more complete, with missing electron density only at the start of N-terminus (Supplementary Fig. S5a, b). The yeast Tom40 structures reported by two groups separately, interestingly, share almost the same missing EM density at the N-terminus, C-terminus, and some regions in the middle of Tom40. Most notably, the missing density of yeast Tom40^G278–P290^(PDB : 6njf) and yeast Tom40^A277-P294^(PDB : 6ucu) both appear to be extended loop structure between the β-strands. Dissimilar to the reported yeast Tom40, the sequence of human Tom40 ends at the 19th β-strand, leaving it without a C-terminal α-helix (Supplementary Fig. S5a, b). In addition, we do not observe a helical segment preceding the internal helix at the N-terminus in our structure, which is consistent with the structural prediction of human Tom40 (Supplementary Fig. S5c). Given that these helices have been proposed to be involved in yeast preprotein translocation^16^, the lack of these segments may indicate potential differences in the translocation mechanism of the human TOM complex.

In the junction between the β-barrel proteins, we observed a phospholipid molecule and identified it to be phosphatidylcholine (PC) by mass spectrometry (Fig. 1c, d, Supplementary Fig. S6). The presence of PC, together with the insertion of the Tom22 helices, contribute to the tilted conformation of the Tom40 β-barrel channels (Fig. 1b, d). The phospholipid molecule interacts with both Tom40s and Tom22s, stabilizing the architectural arrangement of the subunits (Fig. 1f). Phospholipids have been known to be involved in the function of mitochondrial protein complexes associated with protein import, and since PC is the most abundant phospholipid in the mitochondrial membranes comprising ~40% of total phospholipids across yeast and mammalian cells^30,31^, the potential role of PC in the assembly and activity of TOM complex could be an interesting subject for further studies.

As have been reported in the previous studies^14–16^, we observe an α-helical segment that traverses the channel and extends into the IMS (Fig. 1c, e). The α-helix forms a conserved interaction interface with the inner wall of Tom40. In detail, residues T82, N79, P80, H87, and Q97 on the internal part form hydrogen bonds with R195, T200, K253, T264, and Q353 on β strands 7, 8, 11, 12, and 19 of Tom40, respectively (Fig. 1f, Supplementary Fig. S7a). Given its diagonal disposition within the pore, the internal helix could function to provide physical support in the formation of Tom40 β-barrel, providing a structural explanation for previous experimental results that showed hTom40 precursors lacking residues 72–87 are targeted to the mitochondria but do not form stable assembly intermediates^32^. In addition, its presence restricts the cross-sectional diameter of Tom40 to some degree, limiting the sizes of preproteins that could pass through the channel. Most notably, we found that the presence of the internal helix distinctly alters the distribution of electric potential within the pore, detailly discussion would be expanded in the electrostatics part. We, therefore, reason that the internal helix may play an important role in human mitochondrial preprotein translocation. The structural prediction shows that the first ~70 residues at the N-terminus most likely exist in disordered conformation (Supplementary Fig. S5c), consistent with our structural finding. Interestingly, the sequence has an unusually high proline content, whose potential significance deserves further studies.

### Central receptor Tom22

**Fig. 2:**
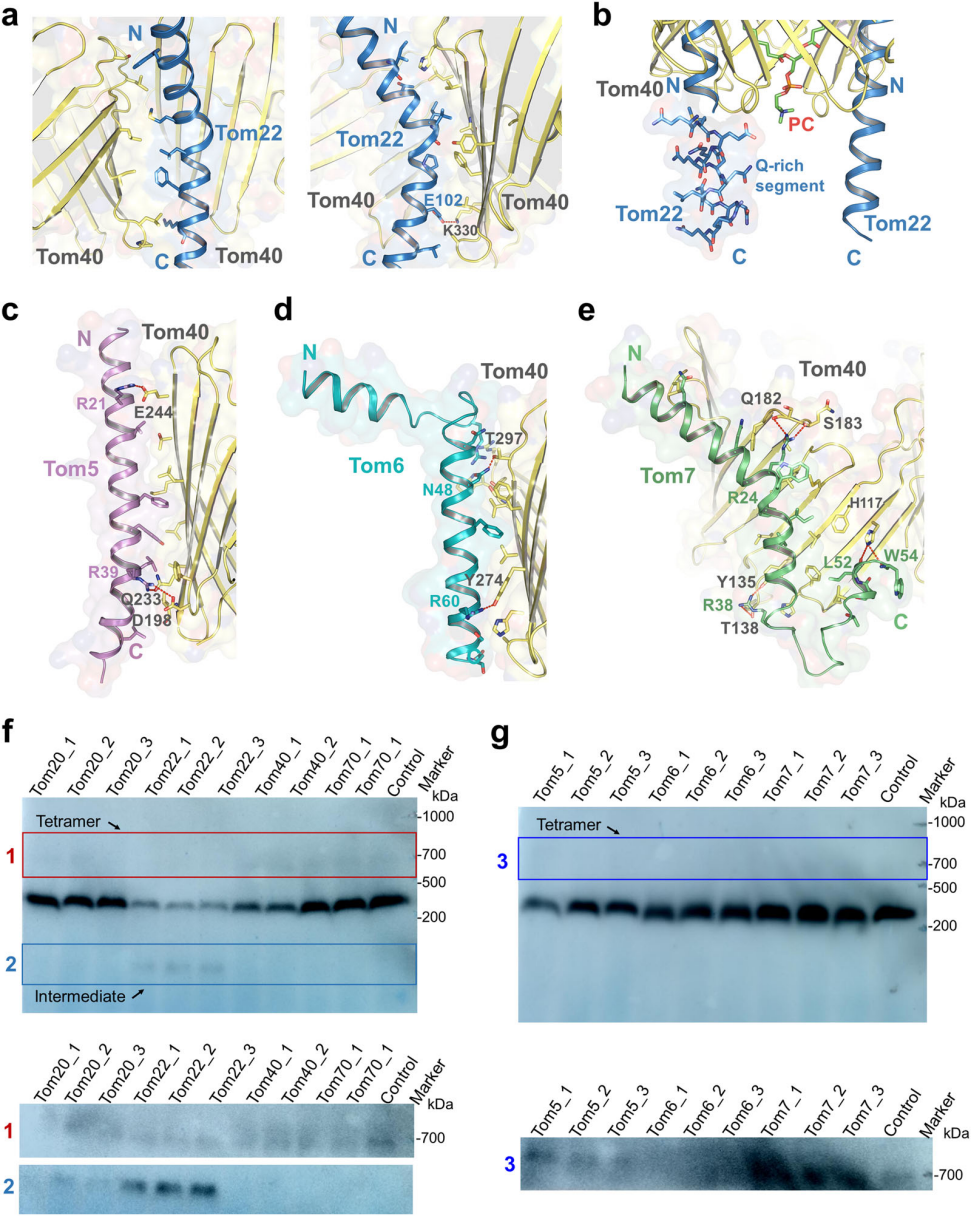
Structure details of Tom-CC and knockdown of TOM subunits. **a** Interactions between Tom22 and Tom40s. The polar interactions are indicated by red dotted lines. Side views are shown. **b** The Q-rich segment (sticks) of Tom22 in IMS. Interactions of Tom40 with Tom5 (**c**), Tom6 (**d**), Tom7 (**e**). The polar interactions are indicated by red dotted lines. **f**, **g** Knockdown of Tom subunits. The tetramer and intermediate state of TOM complex are indicated by boxes, and are separated for a longer exposure as shown in the bottom of each panel. Tom40 was detected with the antibodies against Tom40.

Tom22 is the central receptor of the TOM-CC^33^, inserted between the Tom40 pores along the dimer interface. C-terminally anchored in the OMM by a single transmembrane α-helical segment, it is an amphipathic helix with hydrophilic domains extending into both the cytosol and the IMS^34,35^ (Fig. 1a-d). Its TM segment interacts with the wall of Tom40 through hydrophobic interactions and hydrogen bonding (Fig. 2a). The molecule conforms in a fairly bent shape, with an unusual kink in its helical TM segment, which appears to be caused by a highly conserved proline residue (P98) in the region^34,36^ (Supplementary Figs. S3d, S4d, S7b). The TMD of hTom22 has been suggested by previous experimental results to be involved in both Tom22 integration and regulation of TOM assembly^34^. Notably, dissimilar to the recently reported yeast Tom22 structure^15,16^, the C-terminal half of hTom22 inserts into the IMS at a roughly vertical angle relative to the membrane, whereas its yeast counterpart noticeably tilts toward one side. The resulting proximity of hTom22 with both Tom40 pores enables it to contact the two β-barrel proteins, with both interfaces being relatively conserved (Fig. 2a, Supplementary Fig. S7b), indicating a potentially different role of hTom22 in complex stabilization and function. In addition, a unique segment rich in glutamine residues (Q107–Q118) was observed on the IMS part of hTom22 in our structure (Fig. 2b). A similar segment, termed the Q-rich motif, is present in hTom20 and has been shown previously to be important for preprotein binding and import^37,38^. Furthermore, the IMS domain of hTom22 lacks the acidic character of the fungal receptor^5^, suggesting potential differences in their functional mechanisms.

Previous studies have reported that the assembly of hTom40 is dependent on the levels of free hTom22^32^. To verify the link between the subunits, we performed knockdown of Tom22, which, in agreement with the report, resulted in an increase in TOM intermediate (Fig. 2f). As for the role of Tom22 in preprotein recognition, a specific region on the resolved helix of hTom22 (residues 63–82) has been indicated to be essential for presequence binding^35^. Additionally, previous studies have suggested that the cytosolic domain of Tom22 could be recognized by Tom20^5,39^. Our CL-MS data revealed that hTom20 could contact hTom22 via multiple forms (Supplementary Fig. S8a, b and Table S2). Nevertheless, we obtained samples containing a considerable amount of Tom20 together with the TOM-CC subunits during purification, as was confirmed by SDS-PAGE, BN-PAGE, and MS results. However, we did not observe Tom20 in the resolved structure, likely due to the relatively weak interactions between Tom20 and the core complex^12^. In addition, knockdown of Tom20 in our experiment resulted in weak disassembly of Tom40 from the TOM complex, similar to the effect of Tom22 knockdown (Fig. 2f), which may also be related to the interaction between Tom20 and Tom22^39,40^. Considering that both hTom22 and hTom20 could function as receptors^13,38,41^, a more careful analysis of the link between the two subunits could be an interesting subject for further investigation.

### Small subunits surrounding Tom40 β-barrels

Three distinct single-spanning α-helical densities were observed to surround the Tom40 pore and are assigned to be small Tom proteins Tom5, Tom6, and Tom7 (Fig. 1a–d). All of the three small subunits interact with the β-barrel mostly through hydrophobic interactions, with hydrogen bonding at several locations (Fig. 2c–e). Surface conservation models of Tom40 and the small Tom subunits show that the various interaction surfaces are more evolutionarily conserved than the lipid bilayer-facing surfaces, subtly verifying the reliability of the interactions (Supplementary Fig. S7b–e). In addition, similar to the kink of Tom22, the kink in the helical TM segment of Tom7 appears to be caused by the presence of a universally conserved proline residue (P29) in the region^36^ (Supplementary Figs. S4c, d, S7e). Notably, the proline residue has been shown by a previous study to play a crucial role in the efficient targeting of Tom7 to the outer membrane^36^. The kinks in both Tom7 and Tom22 allow for more contact between the α-helices and the β-barrel, providing structural evidence for the previous proposal that these proline residues help with the integration of α-helical subunits onto the surface of Tom40^34,36^.

The small Tom proteins have long been reported to help with complex stability and assembly^42–44^. Interestingly, hTom7 has been shown to have a significant role in the stabilization of the TOM complex over hTom5 and hTom6, which is notably different from the case in yeast where Tom6 has the primary role^6,42^. Notably, it was reported that hTom7 is the first subunit to associate with Tom40, and through interaction with receptor Tom22, mediate the assembly of the TOM complex^43^. As could be seen structurally, the cytosolic halves of the Tom7 and Tom22 helices sway in the same clockwise direction, and in addition, Tom22 has a helical segment at the N-terminus that bends from the main section and extends toward Tom7 (Fig. 1a-d, Supplementary Fig. S3d), suggesting that the interaction between the subunits might take place on the cytosolic side. Tom5 has been reported in yeast to be involved in preprotein translocation^16^, which is closely related to the proximity of its C-terminal end to the N-terminal β-helix of Tom40, which extends into the IMS. However, hTom40 does not have a helical segment as such preceding the internal helix (Supplementary Fig. S5), indicating the potential difference in regional translocation mechanism across organisms.

Our knockdown experiment showed that the knockdown of Tom6 decreased the level of tetramers (Fig. 2g), consistent with our structural interpretation that Tom6 might mediate the association of dimers into a tetramer (expanded discussion in later tetramer section). However, it is worth noting that the knockdown of Tom7 seems to increase the level of tetramer (Fig. 2g). The role Tom7 played in the assembly of tetramer needs further study. Interestingly, we detected an unassigned density between Tom6 and Tom22 in our map (Fig. 1b, Supplementary Fig. S8d), which appears to be a short peptide, but could not belong to Tom6. We deduce that it likely belongs to Tom22 as our CL-MS results show that residue K141 at the C-terminus of Tom22 crosslinked to K309 of Tom40 (Supplementary Fig. S8b and Table S2). The density could be roughly fitted by the C-terminal sequence of Tom22 and would provide structural support for the crosslinking. However, the results are not sufficient to conclusively determine whether the density belonged to Tom22 or some other protein. Either way, the potential insight into human mitochondrial preprotein translocation the subject brings deserves attention and further investigation.

### Electrostatics of human TOM-CC

**Fig. 3:**
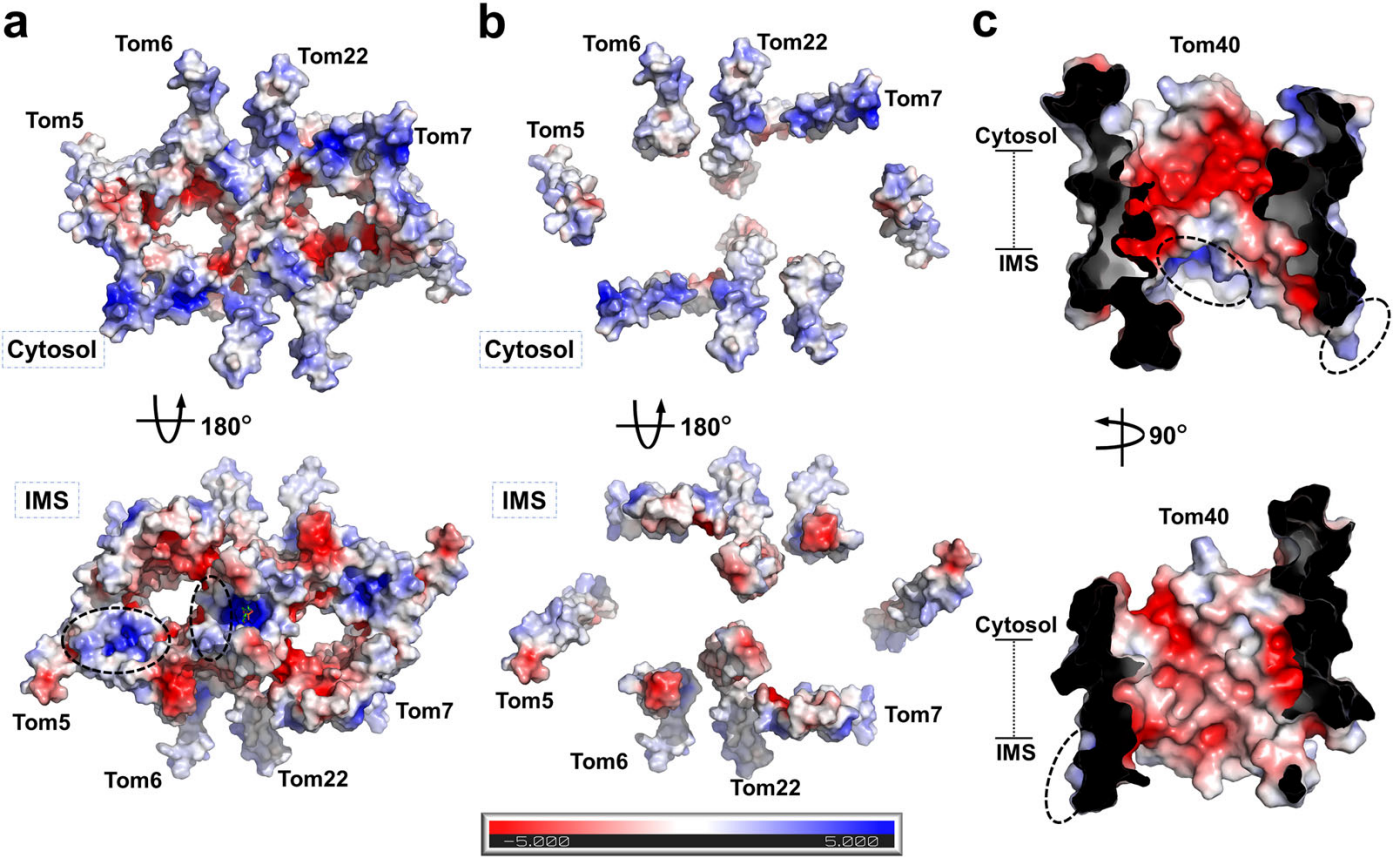
Surface electrostatics of TOM-CC. **a** Surface electrostatic potential of TOM-CC. The periphery and center positive potential regions are indicated with dotted cycles. **b** Surface electrostatic potential of helical Tom subunits. **c** Surface electrostatic potential of Tom40 internal barrel. The periphery and center positive potential regions are indicated with dotted cycles. Shown are cutaway side views.

As briefly discussed above, the subunits of the TOM complex possess notable electrostatic features (Fig. 3, Supplementary Fig. S9), which take in consideration with charged presequences of preproteins and charged regions of other polypeptides or resident proteins, likely play a role in facilitating the translocation of mitochondrial preproteins. The surface electrostatic analysis shows the inner surface of Tom40 to be mostly of negative potential, except a distinctly positive patch at the periphery of the dimer on the IMS side (Fig. 3c, Supplementary Fig. S9b). Remarkably, this positive patch and the especially negative region on the pore lining near the cytosolic side both are in the immediate vicinity of the IMS- and cytosol-facing sides of the internal helix, respectively (Supplementary Fig. S9b). Also, the outer surface of Tom40 has a patch with pronounced positive potential on the IMS side along with the dimer interface (Fig. 3a). The α-helical subunits have uniformly positive potential in the cytosol and negative potential in the IMS, which interestingly switch the overall surface potential of the complex on the cytosolic side and enhance the already existing pattern on the IMS side (Fig. 3a, b, Supplementary Fig. S9a). The unique electrostatic features of hTOM-CC might provide insight into human mitochondrial preprotein translocation.

Research in yeast has revealed two distinct exit sites of the TOM complex for different classes of preproteins^16^, whose relative locations coincide with the two regions of positive potential observed on the IMS side at the periphery and center of the dimer in our structure. Our CL-MS data showed that both preproteins and transporters in the IMS tend to crosslink with Tom40^K309^ and Tom40^K330^, which are parts of the sequences that constitute the central positive region (Tom40^L307–N311^ and Tom40^K330–L337^, respectively); also, a conserved residue Tom40^K253^, located at the positive region around Tom5, is also a multi-crosslinked site (Fig. 3a, Supplementary Figs. S7a, S8b and Table S2). The results suggested that the periphery and center positive potential regions of the hTOM complex might be part of distinct preprotein translocation pathways, similar to its yeast homolog.

However, unlike yeast, further analysis suggests that the exit site might be used for varying classes of preproteins in human. VDAC3^K12^^/^^K15^, VDAC3^K91^, and VDAC3^K18^ were detected to crosslink with Tom40^K309^, Tom40^K90^, and Tom22^K105^, respectively (Supplementary Fig. S8c and Table S2). The implicated VDAC proteins should be in their precursor form, given the crosslinked positions. β-barrel preprotein translocation is reported in yeast to take place at the peripheral site^16^, which diverges from our crosslinking results that seem to suggest that β-barrel preproteins in human take the central pathway. Taken together, with the structural differences observed between yeast and human TOM-CC, we cautiously speculate that the import pathway for β-barrel preproteins is not entirely conserved between the organisms.

### Tetrameric TOM complex

**Fig. 4:**
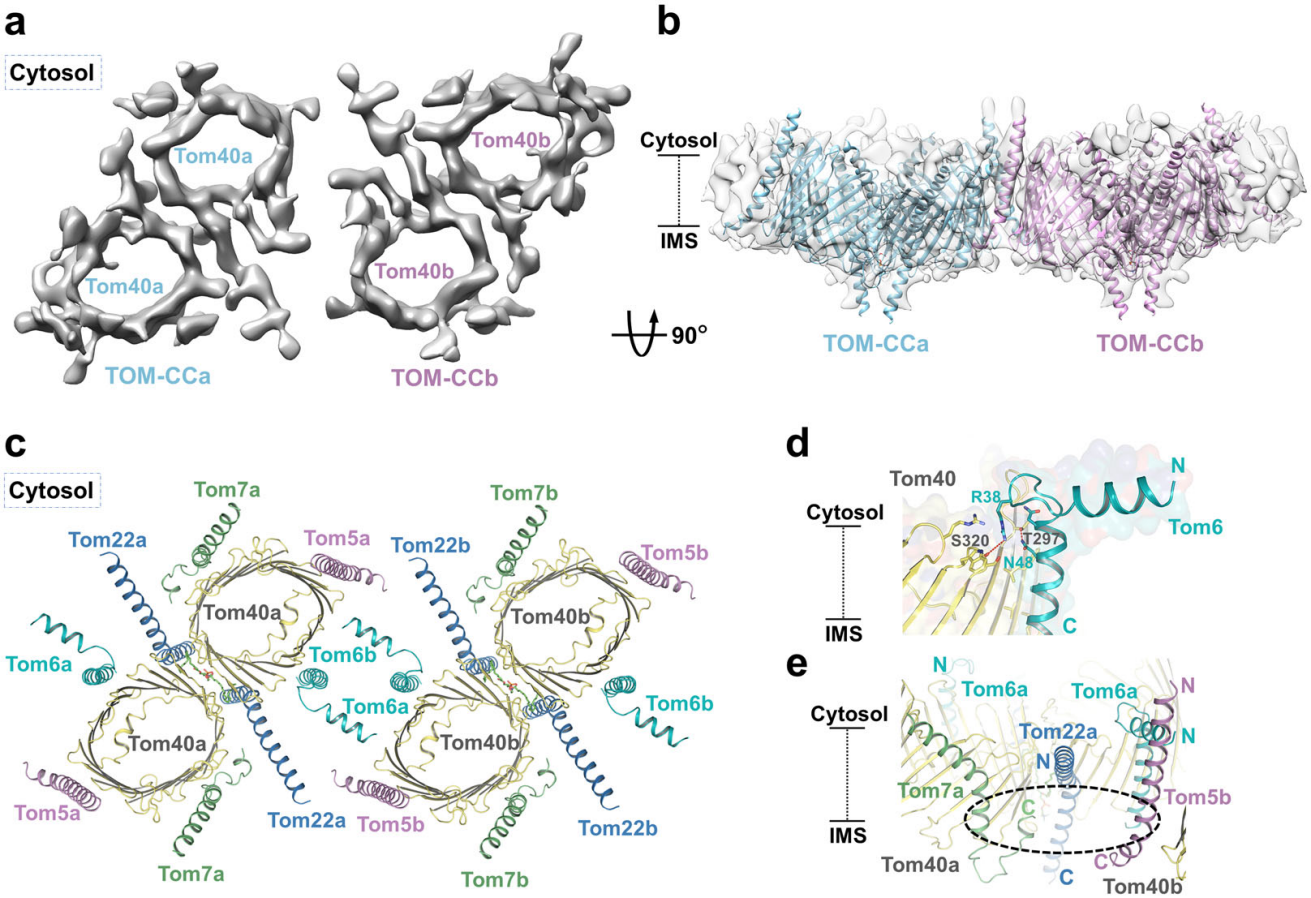
Structure of the tetrameric TOM complex. **a** A 8.5-Å-resolution cryo-EM reconstruction of the tetrameric TOM complex. Two dimeric units are indicated by a and b. Cytosol views are shown. **b** Dimeric TOM complex models fitted in the map. Two dimeric units are indicated by a and b. Side views are shown. **c** Fitted model of tetrameric TOM complex. Cytosol views are shown. **d** Interactions between Tom6^F34–R42^ and Tom40. The polar interactions are indicated by red dotted lines. Side views are shown. **e** A potential binding pocket formed by Tom22, Tom7 from one dimer and Tom5 from the other dimer. The potential pocket is indicated with dotted cycles. Side views are shown.

Through further data collection and particle classification, we surprisingly observed a larger species of TOM complex in tetrameric form and determined its structure to ~8.5 Å (Fig. 4a, Supplementary Fig. S1, 2). Notably, a similar TOM structure of such an oligomeric state has also recently been reported in yeast^15^. Since our map was not of sufficient quality for model building, we fitted the dimeric model into the density map, resulting in a relatively close fit (Fig. 4b, Supplementary Fig. S10a). The dimers arrange in a slightly misaligned positioning, in an overall shape similar to that of a parallelogram, with the cytosolic helices of Tom6s mediating their association (Fig. 4c). Notably, the Tom6 densities in the map appear in closer proximity to each other than represented by the fitted model; moreover, the loop (Tom6^F34–R42^) allows Tom6 to possibly undergo a conformational change in the formation of the tetramer (Fig. 4a, d). The interactions between the cytosolic helices postulated by the model provide a structural explanation for previous functional results that knockdown of Tom6 decreased the level of tetramers (Fig. 2g). The tilted configuration of the cytosolic halves of Tom7 and Tom22, to some degree, flattens the lateral side of the complex, spatially supporting the formation of the binding interface.

In comparison to the reported yeast tetrameric structure, the arrangement of the dimeric units in human tetrameric TOM appears to be less compact, with each subunit given more space to extend along with the interface. In the yeast structure, the region around the two Tom6 subunits seems especially crowded, considering the Tom6s have missing densities at the N-terminus, and the Tom40s have a portion of unresolved sequence between the β-strands in the region. The difference in packing of the dimeric units may allude to possible divergence in the functional mechanism of the complex between organisms in higher oligomeric states. Additionally, the complex in its tetrameric form would physically allow subunits from different dimers to work in coordination with each other, in particular putting Tom7 and Tom22 in proximity to Tom5 from another dimer (Fig. 4c, e). It should be noted that trimeric TOM structure has also been reported in yeast^16,45,46^, which might suggest that the various oligomeric states of TOM might be an adaptive adjustment made by organisms to suit their independent needs for translocation of diverse classes of preproteins.

## Discussion

Despite the overall structural homology human TOM complex demonstrates with its resolved yeast homologs^14–16^, modest structural details are shared among the subunits between the organisms, with the most notable differences in Tom40, Tom22, and Tom6 (Fig. 2a, d, Supplementary Figs. S4b, d, e, S5). The PC molecule observed in the structure contributes to the tilted conformation of the Tom40 pores and the structural integrity of the complex (Fig. 1f). Moreover, the positively charged choline group of PC extends into the IMS and enhances the regional positive charge contributed by the surfaces of Tom40s and Tom22s (Fig. 3a), suggesting that it could have a potential role in the activity of the TOM complex. The α-helical subunits of the complex possess distinct features, which, in addition to their spatial arrangement, could be provided with an explanation when examining the tetrameric form of the complex.

The human TOM complex displays notable electrostatic features, whose apparent specificity likely plays a role in facilitating preprotein transport. The inner surface of the Tom40 channel is mostly of negative potential, except a positive patch on the IMS side at the periphery of the dimer (Fig. 3, Supplementary Fig. S9). Both the positive patch and the especially negative region on the pore lining are in the immediate vicinity of the internal helix (Supplementary Fig. S9b), leading us to speculate that the internal helix likely interacts with preproteins to facilitate their specific passage. The complex has another positive region on the IMS side at the center of the dimer contributed by the surfaces of Tom40s and Tom22s (Fig. 3a). The peripheral and central positive regions likely serve as exits sites for distinct preprotein translocation pathways. The α-helical subunits, notably, expose uniformly positive potential in the cytosol and negative potential in the IMS (Fig. 3a, b, Supplementary Fig. S9). The clear pattern seems to suggest that the small Tom proteins could also, in some way, be involved in preprotein import.

In addition to the dimeric form, we observed the TOM complex in its tetrameric state (Fig. 4a, Supplementary Fig. S10a). Tom6 is shown both structurally and experimentally to be important in the association of two dimers (Figs. 2g, 4a–d). The tilted configuration of the cytosolic halves of Tom22 and Tom7 caused by the kinks spatially enables the formation of the binding interface (Fig. 4a, c, Supplementary Fig. S10). The tetrameric form physically allows subunits from different dimers to work in cooperation, in particular, Tom22 and Tom7 from one dimer are in the proximity of Tom5 from the other dimer, forming a potential binding pocket where preproteins and other mitochondrial outer membrane proteins could dock (Fig. 4e), possibly providing reasoning for the rather dispersed arrangement of small Tom proteins around the Tom40 pore. However, this is merely an interesting model, whether the molecules cooperate or not in the context of tetramer needs to be verified by further study. In addition, the tetrameric arrangement blurs the boundary between the two proposed exit sites of the TOM complex, bringing the peripheral site of one dimer close to the central site of the other (Supplementary Fig. S10b), which could improve the efficiency of preprotein translocation. Interestingly, the trimeric TOM complex has also been reported in yeast^16,45,46^. The tetrameric form we observe here may be an intermediate state of the trimer or vice versa. Alternatively, the various oligomeric states of the complex could be evolutionary adjustments organisms have made to suit their individual needs for mitochondrial preprotein import.

**Fig. 5:**
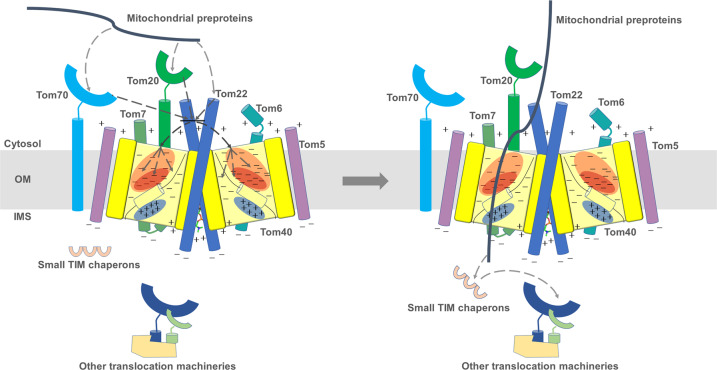
Model of preproteins import through TOM complex. The preproteins are recognized and guided to the Tom40 pores by Tom20, Tom70, and Tom22, where the electrostatics of the complex further distinguish different classes of preproteins and lead them to separate pathways within the channel. Once inside, the entry of preproteins would be further regulated by the internal helix of Tom40. Upon passing through the protein conducting channel, the disordered N-terminal of Tom40 and the IMS domain of Tom22, together with the small TIM chaperones in the vicinity of the positively charged regions of Tom40, facilitate the transfer of preproteins to other protein translocation machineries in the mitochondria.

The high-resolution structure of the human TOM complex provides insight into human mitochondrial preprotein translocation and possible ways its mechanism may differ from that of its yeast counterpart. Based on our research and previous studies of the TOM complex^3,9,47^, we propose the following mechanism for human mitochondrial protein translocation: Tom20, Tom70, and Tom22 independently or collectively recognize the preproteins and guide them to the Tom40 pores, where the electrostatics of the complex further distinguish different classes of preproteins and lead them to separate pathways within the channel. Once inside, the entry of preproteins would be further regulated by the internal helix of Tom40, which is potentially flexible in vivo and could adjust the physical size and inner electrostatics of the Tom40 barrel. Upon passing through the protein conducting channel, the disordered N-terminal of Tom40 and the IMS domain of Tom22, together with the small TIM chaperones in the vicinity of the positively charged regions of Tom40, facilitate the transfer of preproteins to other protein translocation machineries in the mitochondria (Fig. 5). The tetrameric TOM complex might enable the subunits from different dimers to collaborate, possibly linking the two proposed pathways, improving the efficiency of human mitochondrial preprotein transport.

## Supplementary information

Supplementary Information

## Data Availability

The atomic coordinates of human TOM-CC have been deposited in the Worldwide Protein Data Bank with the accession code 7CK6, respectively. The corresponding maps have been deposited in the Electron Microscopy Data Bank with the accession code EMD-30382, respectively.

## Supplementary information

**Supplementary Information** accompanies the paper at (10.1038/s41421-020-00198-2).
