# Supplementary material for: Atomic structure of human TOM core complex
Source: Cell Discov. 2020 Sep 29;6:67. doi: 10.1038/s41421-020-00198-2 (PMC7522991; doi:10.1038/s41421-020-00198-2)
Supplement: Supplementary file 1 — Supplementary Information [file 41421_2020_198_MOESM1_ESM.pdf]

Materials and Methods

Transient coexpression of human TOM complex

The optimized coding DNAs for human TOMM40 (Uniprot: O96008), TOMM22 (Uniprot: Q9NS69), TOMM7 (Uniprot: Q9P0U1), TOMM6 (Uniprot: Q96B49) and TOMM5 (Uniprot: Q8N4H5) were cloned into the pcDNA3.1(-) vector, with tandem twin Strep-tag at the C terminus of TOMM22. The HEK293F cells (Invitrogen) were cultured in SMM 293T-I medium (Sino Biological Inc.) supplemented with 8% CO_2 in a ZCZY-CS8 shaker (Zhichu, 120 r.p.m.) at 37°C. When density reached 2 × 106 cells per ml, the cells were transfected with the plasmids encoding TOMM70, TOMM40, TOMM22, TOMM20, TOMM7, TOMM6 and TOMM5, incubate with Polyethylenimine (PEI) for 15 minutes at a mass ratio of 1:3. Transfected cells were cultured for 48 hours before harvesting.

Purification of the TOM complex

All procedures are carried out at 4°C. Four liters of transfected cells were harvested, washed with 1X PBS and resuspended in 25 mM Tris pH 7.8, 150 mM NaCl, and 1mM PMSF. The suspension was lysed by sonication for 5 min, then was further centrifuged at 20,000xg for 45 min to obtain the membrane. The pellet was suspended and extracted in 25mM Tris pH 7.8, 150mM NaCl with 1% digitonin. After incubation for 2 hours, the extraction was centrifuged at 20,000xg for 25 min at 4°Cand the supernatant was applied to Strep-Tactin Sepharose (IBA) by gravity at 4°C. The resin was washed three times with the W buffer, which contains 25mM Tris pH 7.8, 150mM NaCl, with 0.1% digitonin. The target proteins were eluted with W buffer plus 5mM desthiobiotin (Sigma), concentrated to 100ul by 100 kDa cut-off centrifugal filter (Millipore) and further purified by Superose6 5/150 GL (GE Healthcare) also in the W buffer. The peak fractions were collected for EM sample preparation. The presence of the complex was verified by SDS-PAGE, BN-PAGE and confirmed by mass spectrometry (Supplementary Fig. S1a-c).

Blue native PAGE

Following the Blue native PAGE protocol[1], the purified TOM core complex was subjected to 3-10% blue native PAGE mini gel (1.5 × 8.3 × 7.3mm） for native electrophoresis at 150V for 4h (Fig. 2g-f, Supplementary Fig. S1c).

Cryo-electron microscopy

4-µL aliquots of digitonin-solubilized TOM-CC at a concentration of 4.5 mg/ml were applied to discharged 400-mesh Quantifoil R1.2/1.3 grids (Quantifoil, Micro Tools GmbH, Germany). Grids were blotted for 3.5 sec and plunged into liquid ethane using an FEI Mark IV Vitrobot operated at 4 °C and 100% humidity. Micrographs were collected on a Titan Krios microscope operated at a voltage of 300 kV with a Gatan K2 Summit direct electron detector. Images were recorded using a 300 kV Titan Krios electron microscope with serialEM software (Thermo Fisher) at a nominal magnification of 130,000 × using super-resolution mode. The pixel size was 1.08 Å/pixel and the defocus range were set from -1.3 µm to -2.3 µm. The total dose rate on the detector was about 50 e/ Å2 with a total exposure time of 8 s. Each micrograph stack contains 32 frames. Four batches of data were collected with two cryo-samples, obtaining 5,908 micrographs in total.

Each micrograph was corrected for sub-region motion correction and dose weighting using UCSF MotionCor2[2]. Gctf was used to determine the contrast transfer function (CTF) parameter and produce the CTF power spectrum on basis of summed micrographs from MotionCor2 for all micrographs[3]. The 5,443 CTF-corrected cryo-EM images were manually selection. Particles were auto-picked on micrographs with dose-weighting using RELION[4]. Briefly, about 1,000 particles were manually picked from a subset of images and extracted in a box size of 200 pixels and a mask diameter of 200 Å. Extracted particles were subjected to 2D classification requesting 10 classes, 8 classes of which showed representative views and were selected as templates for automated particle picking. The resulting 2D averages were served as the templates for particle auto-picking, 1,638,577 particles picked from 5,443 images. For the dataset, particle selection, 2D and 3D classifications were performed on a binned dataset with a pixel size of 5.4 Å using RELION, which were then manually inspected to exclude noise and other bad particles. Two rounds of 2D classification requesting 100 classes were resulted in 823,450 particles, and two rounds of 3D classification which contained 408,208 particles and 30,916 dimeric TOM-CC like particles. An initial model was generated from 20,000 best particles using cryoSPARC ab initio reconstruction requesting five classes with C1 symmetry[5]. A total of 347,796 particles from these 3D classes was re-extracted to the original pixel size of 1.08 Å, and classified into five classes with C1 symmetry using a reference model generated from cryoSPARC, which had been low-pass filters to 20 Å[5]. Two rounds of 3D classification and the most populated class containing 159,369 particles was subjected to further 3D auto-refinement with

C2 symmetry. The refinement resulted in an overall structure at a resolution of 3.91 Å, which allowed initial model building. To further improve the resolution, we performed CTF refinement, which yielded a map at 3.43 Å resolution.

The 30,916 tetrameric TOM complex like particles performed two rounds 2D classification and one round 3D classification which obtained 11,184 good particles. After re-extracted to the original pixel size of 1.08 Å, 11,184 particles were subjected to further 3D auto-refinement with C2 symmetry. The global resolution for tetrameric TOM complex was 8.53 Å. All reported resolutions are based on the gold-standard Fourier Shell Correlation (FSC) = 0.143 criteria[6], and the final FSC curve was corrected for the effect of a soft mask using high-resolution noise substitution. Final density maps were sharpened by B-factors calculated with the RELION post-processing program. All reported resolutions are based on the gold-standard FSC = 0.143 criteria, and the final FSC curve was corrected for the effect of a soft mask using high-resolution noise substitution. The local resolution map was calculated using ResMap[7] (Supplementary Fig. S1d-e, 2, Table S1).

Quantification and Statistical Analyses

After refinement, map CC between model and EM map was 0.66, indicative of a reasonable fit at the present resolution. The resulting model was also used to calculate a model-map FSC curve, which agreed well with the gold-standard FSCs generated during the RELION refinement. The final model has good stereochemistry, as evaluated using MolProbity[8].

Model Building, Refinement and Validation

Atomic models of the TOM-CC subunits were predicted and modeled using the webserver (http://www.sbg.bio.ic.ac.uk/phyre2) of Phyre2[9]. We assigned homology models of the TOM-CC subunits, Tom40, Tom 22, Tom7, Tom6 and Tom5 into our density maps. The models were further optimized by Coot[10]. For cross-validation against overfitting, we randomly displaced the atom positions of the final model by up to a maximum of 0.5 Å, and refined against the half map generated by RELION 3D auto-refine procedure, resulting a model named Test. Then we calculated the FSC curve of both half map against the model Test and compared with the FSC curve of the final model against the summed map generated by RELION 3D auto-refine procedure. To build the human tetrameric TOM complex models, we fitted the density maps of TOM-CC into the 8.53 Å tetramer

map and combined these part maps into a whole map in UCSF Chimera. Based on the combined map, the human tetramer TOM complex model was built. And then, the model with the ligands were subjected to global refinement and minimization in real_space_refinement using PHENIX. All ligands and phospholipids models were generated using elbow[11] module in PHENIX[12] by their geometric constraints. The phospholipid was docked into densities and refined in COOT. All the figures were created in PyMOL[13], Chimera and UCSF Chimera(X)[14], quantitative electrostatics were calculated using APBS[15], and conservation levels were generated with ConSurf[16], sequences alignment of Tom subunits were generated by the ESPript[17], the structure predictions of human & yeast Tom subunits are based on the Predict Secondary Structure (PSIPRED)[18].

Cell Culture and Transient knockdown of Tom subunits

Hela (Thermo Fisher Scientific) cells were grown in DMEM media (Corning) supplemented with 10% fetal bovine serum (Thermo Fisher Scientific) and cultured in a 37°C, 5% CO_2 incubator. 4.5x10^5 Hela cells were incubated with corresponding short hairpin RNA (siRNA) at a final concentration of 66nM, and cultured in a 37°C, 5% CO_2 incubator for 48h. RNA was extracted by RNeasy Plus Mini Kit (Qiagen), and cDNA conversion was performed by TransScript OneStep gDNA Removal and cDNA Synthesis SuperMix (Trans) at 42°C for 20min. Quantitative real-time polymerase chain reaction (RT-qPCR) was conducted by the PerfectStart Green qPCR SuperMix (Trans) as the manual recommended. The mRNA expression of Tom5, Tom6, Tom7, Tom20, Tom22, Tom40 and Tom70 were normalized to GAPDH. And the sequences used were as follows:

Tom5, forward: GACCCGGAGGAGATGAAA

Tom5, reverse: AGATAAATGGAGTGACTCGC

Tom6, forward: CTGCTGGCTCGGCTAATGAAA

Tom6, reverse: ATCAGTGGCAAAGCGGTAGAC

Tom7, forward: CATTCGCTGGGGCTTTATCC

Tom7, reverse: TCTGCACCCCTCTTAAATCCC

Tom20, forward: GGTACTGCATCTACTTCGACCG

Tom20, reverse: TGGTCTACGCCCTTCTCATATTC

Tom22, forward: CAGTCCCCGGACGAATTGC

Tom22, reverse: CGACAGGGTCTCATCTAGCTC

Tom40, forward: CAGTACGAGTCGAAGTTCGGA

Tom40, reverse: GCACTCCTCGAATGTGCCC

Tom70, forward: GGTCTTCCGTTCGCTGTTG

Tom70, reverse: CGCGGGTGCCATATACCTG

GAPDH, forward: TGTGGGCATCAATGGATTTGG

GAPDH, reverse: ACACCATGTATTCCGGGTCAAT

Western Blotting

Harvested Hela cells were lysed by 1% digitonin (1% digitonin, 25mM Tris pH=7.4, 150mM NaCl) at 4°C for 30min. After centrifugation at 13,000xg, 4°C for 15min, the supernatant was collected for Blue native PAGE (BN-PAGE) with the acrylamide concentration from 3% to 12%. The separated proteins were electro-transferred to PVDF membranes (Millipore) at 300 mA for 1h. The membranes were incubated with acetic acid for 8min, followed with incubation with methyl alcohol for 5min for 3 times. Tom40 was detected with the antibodies against Tom40 (Proteintech) (Supplementary Fig. S2g-f).

CL-MS analyses and phospholipid identification

Mix BS3 crosslinker solution and the purified protein with a 20-fold excess (20:1 Crosslinker : Protein) so that the final crosslinker concentration is 1.25 mM, 2.50 mM, 3.75 mM, and 5 mM, respectively, incubate for 45 min at room temperature. Quench and unreacted BS3 with 60 mM Tris for 15 min at room temperature. Then, the protein was sampled and separated by SDS-PAGE (Supplementary Fig. S8a). The cross-linked proteins were treated with 5 mM DTT for 60 min and alkylated with 12 mM iodoacetamide for 45 min at room temperature in the dark, followed by trypsin digestion (Promega, # 8V5280) at 37°C overnight. The generated peptides were desalted and fractionated by HPLC into 12 fractions at pH 10 and then analyzed by LC-MS/MS. The acquired RAW data files were searched against a database containing Mitochondrial proteins using both XlinkX search engine integrated as a node in Proteome Discoverer software (Version 2.3)[19](Supplementary Table S2).

To extract the lipids, we add methanol to the purified TOM complex protein solution, shake and centrifuge the mixture to remove the proteins. The supernatant is collected for Matrix-assisted laser desorption/ionization of mass spectrometry (MALDI-MSMS). Data for different types of phospholipids are searched in the Human Metabolome Database (HMDB)[20](Supplementary Fig. S6).

References

1 Wittig, I., Braun, H. P. & Schagger, H. Blue native PAGE. *Nat Protoc* **1**, 418-428, doi:10.1038/nprot.2006.62 (2006).

2 Zheng, S. Q. *et al.* MotionCor2: anisotropic correction of beam-induced motion for improved cryo-electron microscopy. *Nat Methods* **14**, 331-332, doi:10.1038/nmeth.4193 (2017).

3 Zhang, K. Gctf: Real-time CTF determination and correction. *J Struct Biol* **193**, 1-12, doi:10.1016/j.jsb.2015.11.003 (2016).

4 Scheres, S. H. RELION: implementation of a Bayesian approach to cryo-EM structure determination. *J Struct Biol* **180**, 519-530, doi:10.1016/j.jsb.2012.09.006 (2012).

5 Punjani, A., Rubinstein, J. L., Fleet, D. J. & Brubaker, M. A. cryoSPARC: algorithms for rapid unsupervised cryo-EM structure determination. *Nat Methods* **14**, 290-296, doi:10.1038/nmeth.4169 (2017).

6 Scheres, S. H. & Chen, S. Prevention of overfitting in cryo-EM structure determination. *Nat Methods* **9**, 853-854, doi:10.1038/nmeth.2115 (2012).

7 Swint-Kruse, L. & Brown, C. S. Resmap: automated representation of macromolecular interfaces as two-dimensional networks. *Bioinformatics* **21**, 3327-3328, doi:10.1093/bioinformatics/bti511 (2005).

8 Chen, V. B. *et al.* MolProbity: all-atom structure validation for macromolecular crystallography. *Acta Crystallogr D Biol Crystallogr* **66**, 12-21, doi:10.1107/S0907444909042073 (2010).

9 Kelley, L. A., Mezulis, S., Yates, C. M., Wass, M. N. & Sternberg, M. J. The Phyre2 web portal for protein modeling, prediction and analysis. *Nature protocols* **10**, 845-858, doi:10.1038/nprot.2015.053 (2015).

10 Emsley, P. & Cowtan, K. Coot: model-building tools for molecular graphics. *Acta Crystallogr D Biol Crystallogr* **60**, 2126-2132, doi:10.1107/S0907444904019158 (2004).

11 Moriarty, N. W., Grosse-Kunstleve, R. W. & Adams, P. D. electronic Ligand Builder and Optimization Workbench (eLBOW): a tool for ligand coordinate and restraint generation. *Acta Crystallogr D Biol Crystallogr* **65**, 1074-1080, doi:10.1107/S0907444909029436 (2009).

12 Adams, P. D. *et al.* PHENIX: a comprehensive Python-based system for macromolecular structure solution. *Acta Crystallogr D Biol Crystallogr* **66**, 213-221, doi:10.1107/S0907444909052925 (2010).

13 Schrodinger, LLC. *The PyMOL Molecular Graphics System, Version 1.8* (2015).

14 Pettersen, E. F. *et al.* UCSF Chimera--a visualization system for exploratory research and analysis. *J Comput Chem* **25**, 1605-1612, doi:10.1002/jcc.20084 (2004).

15 Baker, N. A., Sept, D., Joseph, S., Holst, M. J. & McCammon, J. A. Electrostatics of nanosystems: application to microtubules and the ribosome. *Proc Natl Acad Sci U S A* **98**, 10037-10041, doi:10.1073/pnas.181342398 (2001).

16 Ashkenazy, H. *et al.* ConSurf 2016: an improved methodology to estimate and visualize evolutionary conservation in macromolecules. *Nucleic Acids Res* **44**, W344-350, doi:10.1093/nar/gkw408 (2016).

17 Robert, X. & Gouet, P. Deciphering key features in protein structures with the new ENDscript server. *Nucleic Acids Research* **42**, W320-W324, doi:10.1093/nar/gku316 (2014).

18 Buchan, D. W. A. & Jones, D. T. The PSIPRED Protein Analysis Workbench: 20 years on. *Nucleic Acids Res* **47**, W402-W407, doi:10.1093/nar/gkz297 (2019).

19 Krzywinski, M. *et al.* Circos: an information aesthetic for comparative genomics. *Genome Res* **19**, 1639-1645, doi:10.1101/gr.092759.109 (2009).

20 Wishart, D. S. *et al.* HMDB 4.0: the human metabolome database for 2018. *Nucleic Acids Res* **46**, D608-D617, doi:10.1093/nar/gkx1089 (2018).

Supplementary figures and Figure legends

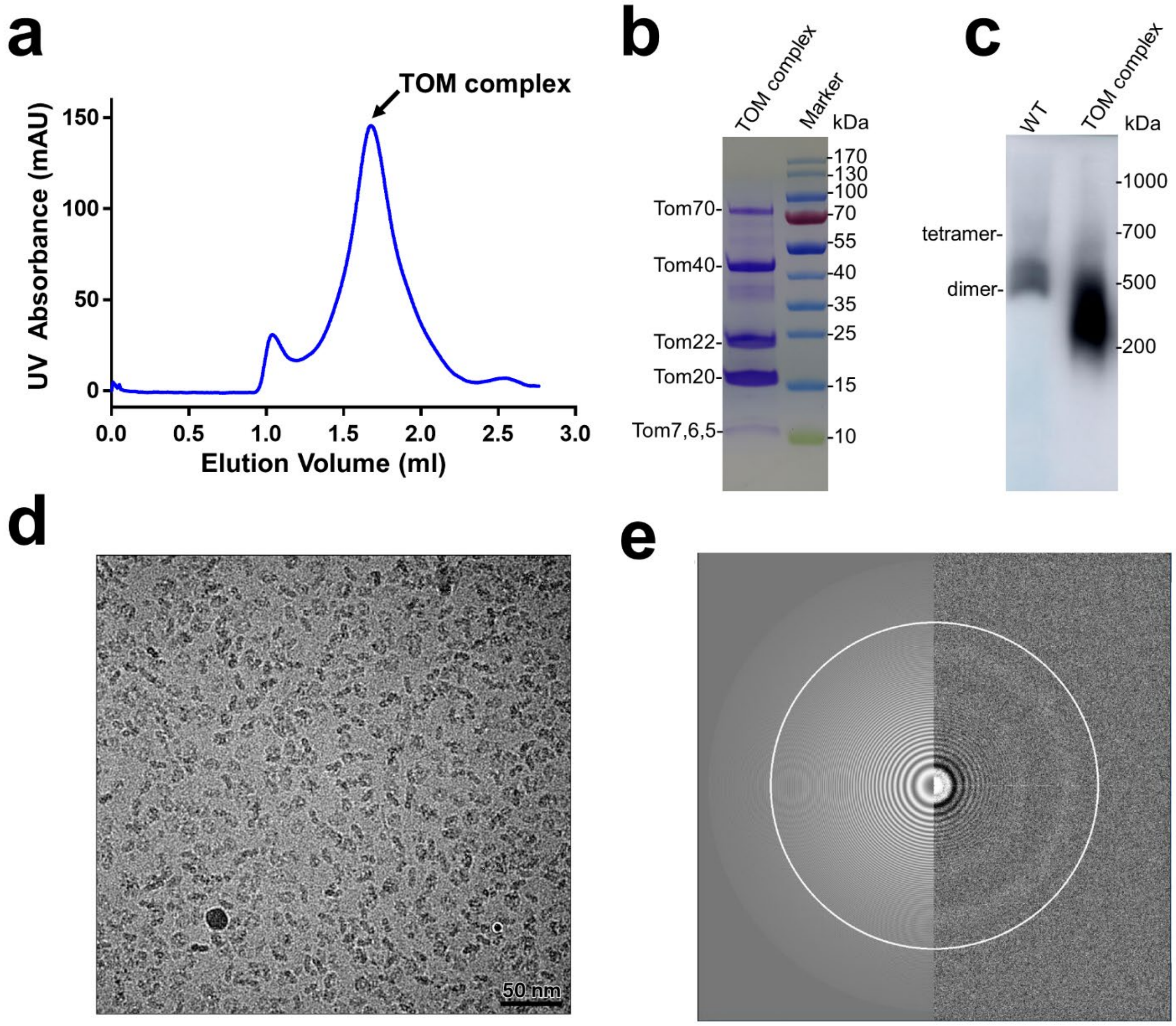

Supplementary Fig. S1 | Biochemical characterization of the TOM complex.

a, A representative trace of size-exclusion chromatography of TOM complex by Superose6 5/150 GL column.

b, SDS-PAGE of purified TOM complex. Band of each subunit was indicated, respectively.

c, Comparison of wild type and purified TOM complex, detected by BN-PAGE. Tom40 was detected with the antibodies against Tom40.

d, A representative cryo-EM micrograph of TOM-CC.

e, Power spectrum of the micrograph in (d).

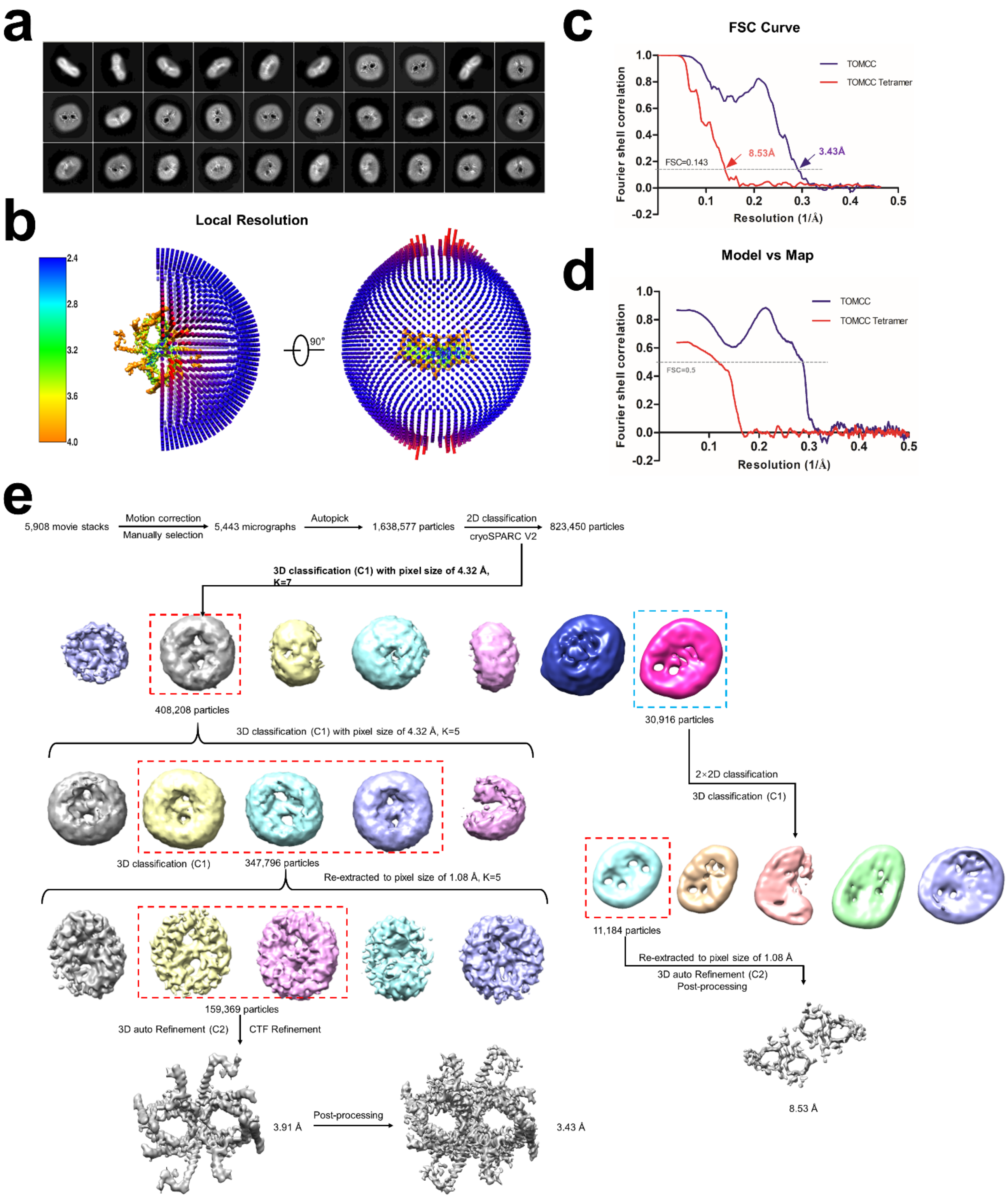

Supplementary Fig. S2 | Cryo-EM analysis of the TOM-CC.

a, 2D class averages of cryo-EM particles.

b, Particle orientation distributions of the TOM-CC in the last iteration of the 3D auto-refinement.

c,d, Gold-standard Fourier Shell Correlation (FSC) curve of the final density maps and cross-validation

with the use of the corresponding maps and models. Dimeric and Tetrameric TOM complex are indicated, respectively.

e, Workflow of 3D classification and refinement of cryo-EM particles. 823,450 particles were kept after 2D classification, and subject to rounds of 3D classification. A final dataset containing 159,396 particles were used for TOM-CC high-resolution refinement, and 11,184 particles were used for tetrameric TOM complex reconstruction (see methods for more details).

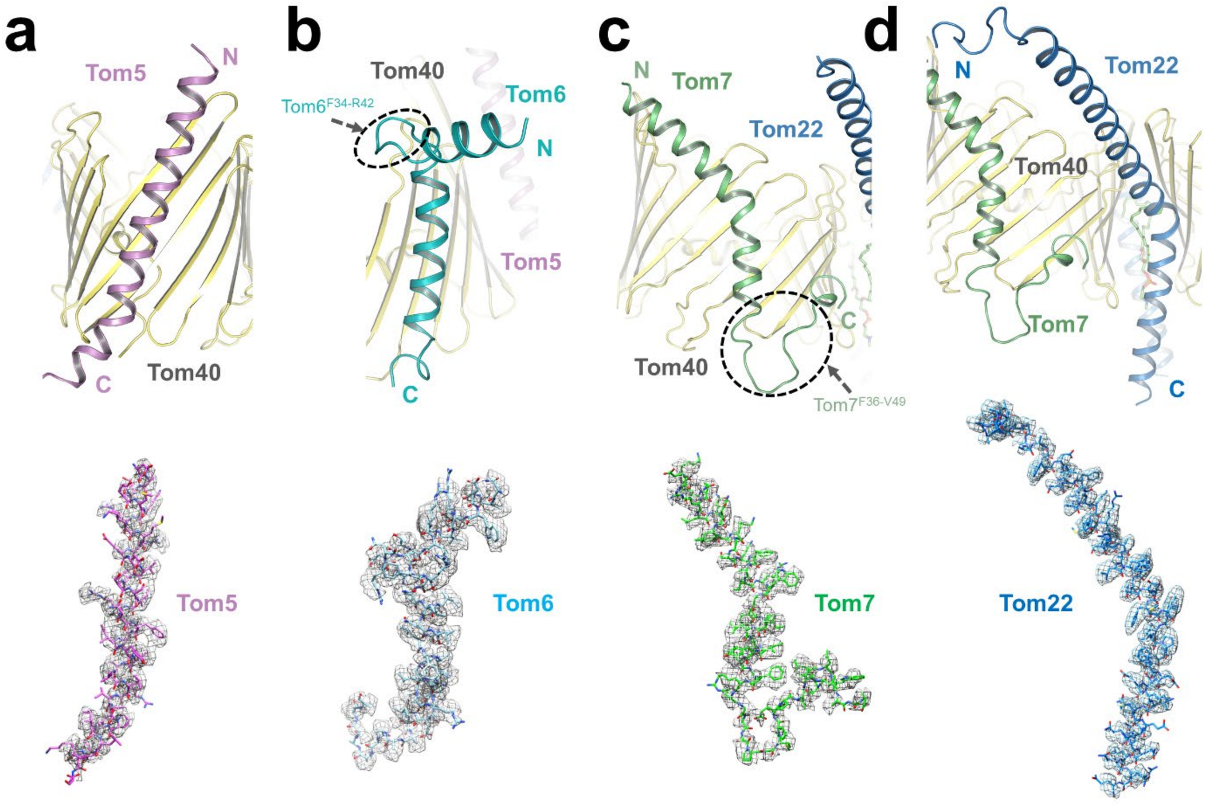

Supplementary Fig. S3 | Overall Structure of helical Tom subunits.

Overall structure of Tom5 (a), Tom6 (b), Tom7 (c), Tom22 (d). Distribution and features of each subunit are shown in up panel and models fitted in map are shown in low panel. The cryo-EM densty of each helical Tom subunit is displayed at 4 σ (Tom5), 4 σ (Tom6), 6 σ (Tom7), and 9 σ (Tom22) contour level, respectively.

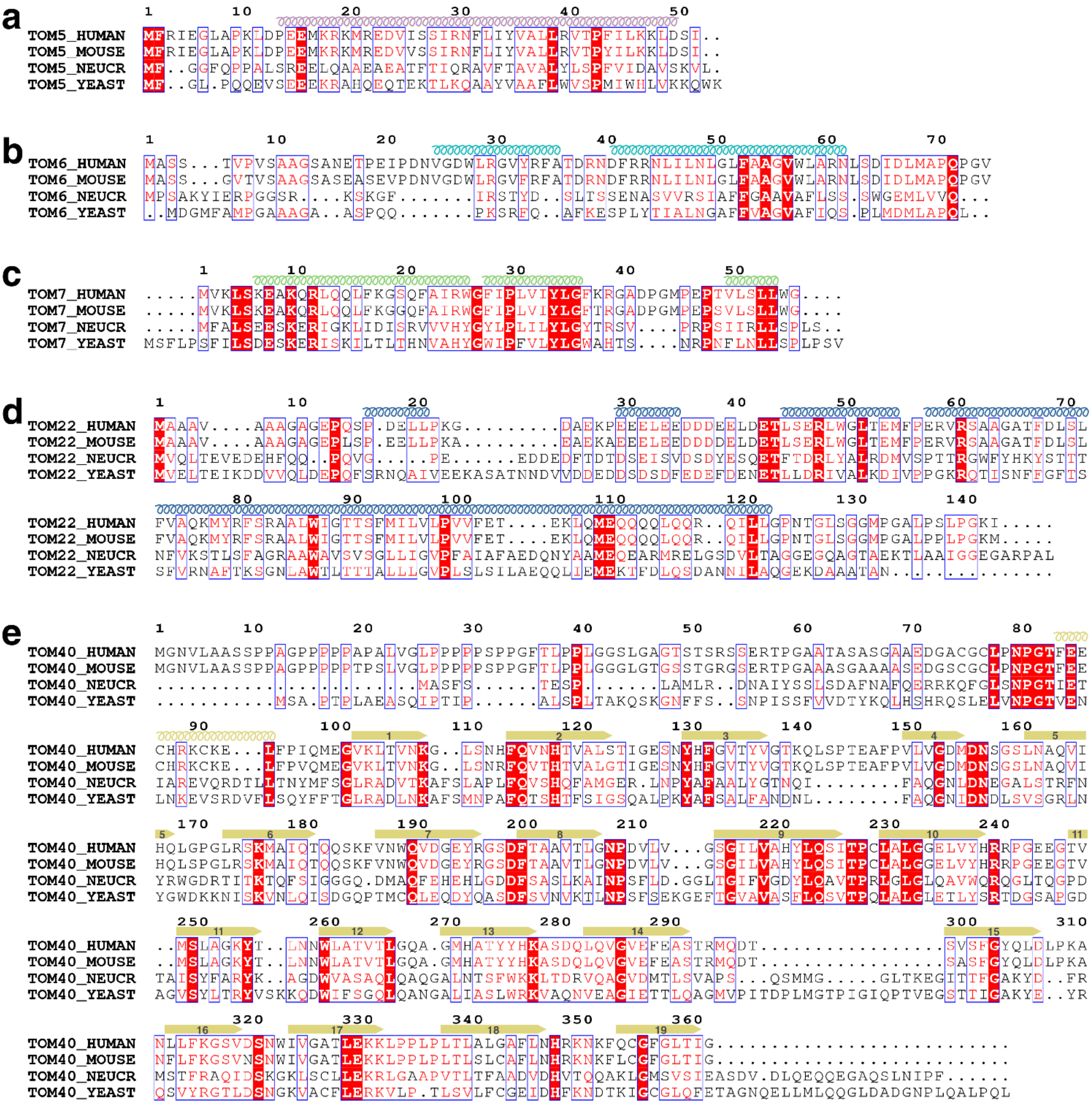

Supplementary Fig. S4 | Sequence comparison of TOM-CC subunits between different species. The threshold value of the alignment is 0.7, which means if the similarity score assigned to a column is greater than this value, residues are considered as highly similar and are colored in red and framed in blue. Secondary structure based on human Tom subunits are indicated. The alignments were generated by the ESPript.

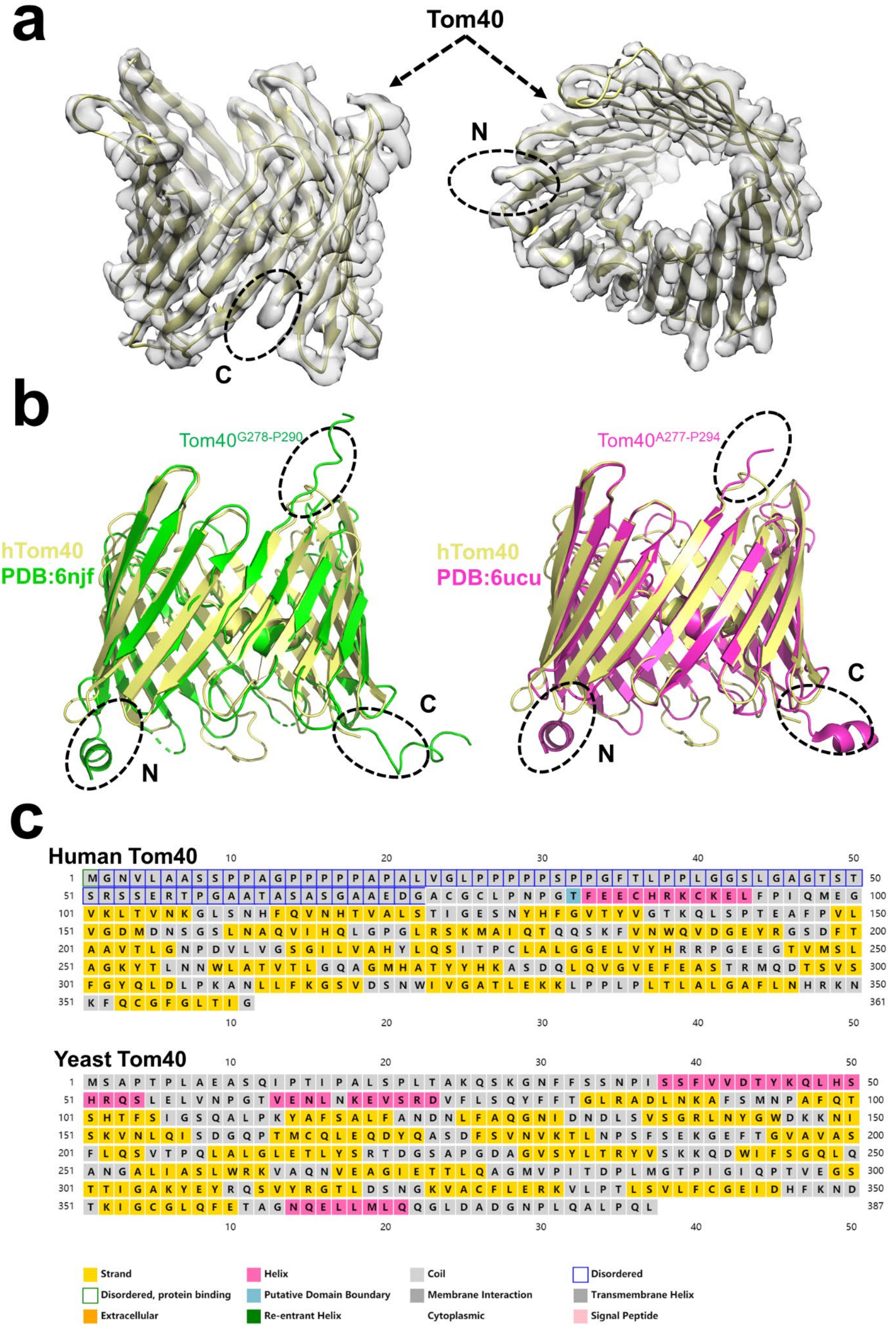
a
Tom40
N
C
b
Tom40G278-P290
Tom40A277-P294
hTom40
PDB:6njf
hTom40
PDB:6ucu
N
C
N
C
c
Human Tom40
1 MGNVLAASSPPAGPPPPPAPALVGLPPPPPSPPGFTLPPLGGSLGAGTST 50
51 SRSSERTPGAATASASGAAEDGACGCLPNPGTFEECHRKCKELFPIQMEG 100
101 VKLTVNKGLSNHFQVNHTVALSTIGESNYHFGVTYVGTKQLSPTEAFPVL 150
151 VGDMDNSGSLNAQVIHQLGPGLRSKMAIQTQQSKFVNWQVDGEYRGSDFT 200
201 AAVTLGNPDVLVGSGILVAHYLQSITPCLALGGELVYHRRPGEEGTVMSL 250
251 AGKYTLNNWLATVTLGQAGMHATYYHKASDQLQVGVEFEASTRMQDTSVS 300
301 FGYQLDLPKANLLFKGSVDSNWIVGATLEKKLPPLPLTLALGAFLNHRKN 350
351 KFQCGFGLTIG 361
Yeast Tom40
1 MSAPTPLAEASQIPTIPALSPLTAKQSKGNFFSSNPISSFVVDTYKQLHS 50
51 HRQSLELVNPGTVENLNKEVSRDVFLSQYFFTGLRADLNKAFSMNPAFQT 100
101 SHTFSIGSQALPKYAFSALFANDNLFAQGNIDNDLSVSGRLNYGWDKKNI 150
151 SKVNLQISDGQPTMCQLEQDYQASDFSVNVKTLNPSFSEKGEFTGVAVAS 200
201 FLQSVTPQLALGLETLYSRTDGSAPGDAGVSYLTRYVSKKQDWIFSGQLQ 250
251 ANGALIASLWRKVAQNVEAGIETTLQAGMVPITDPLMGTPIGIQPTVEGS 300
301 TTIGAKYEYRQSVYRGTLDSNGKVACFLERKVLPTLSVLFCGEIDHFKND 350
351 TKIGCGLQFETAGNQELLMLQQGLDADGNPLQALPQL 387
Strand
Helix
Coil
Disordered
Disordered, protein binding
Putative Domain Boundary
Membrane Interaction
Transmembrane Helix
Extracellular
Re-entrant Helix
Cytoplasmic
Signal Peptide

Supplementary Fig. S5 | Distinct features of human Tom40

a, Model of human Tom40 fitted in map. The N- and C- terminal are indicated with dotted cycles.

b, Alignments of human Tom40 model with yeast Tom40 models PDB: 6njf and PDB: 6ucu, respectively. The distinct differences between models are indicated with dotted cycles.

c, Secondary structure prediction of human and yeast Tom40, respectively. The structure predictions are based on the PSIPRED.

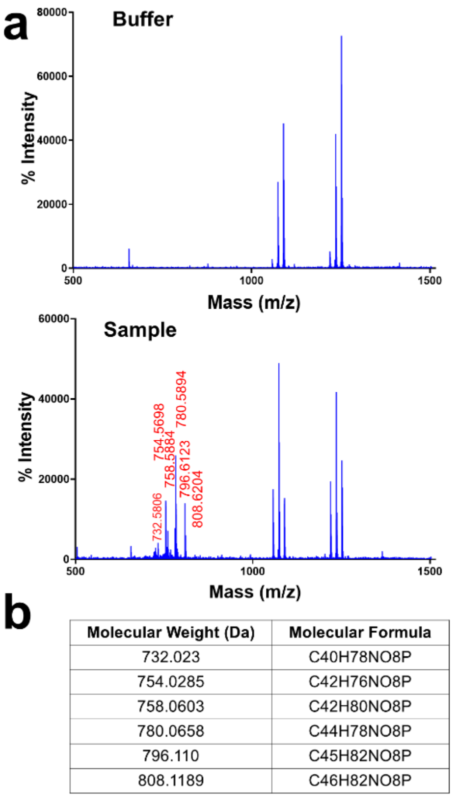

b

Molecular Weight (Da)	Molecular Formula
732.023	C40H78NO8P
754.0285	C42H76NO8P
758.0603	C42H80NO8P
780.0658	C44H78NO8P
796.110	C45H82NO8P
808.1189	C46H82NO8P

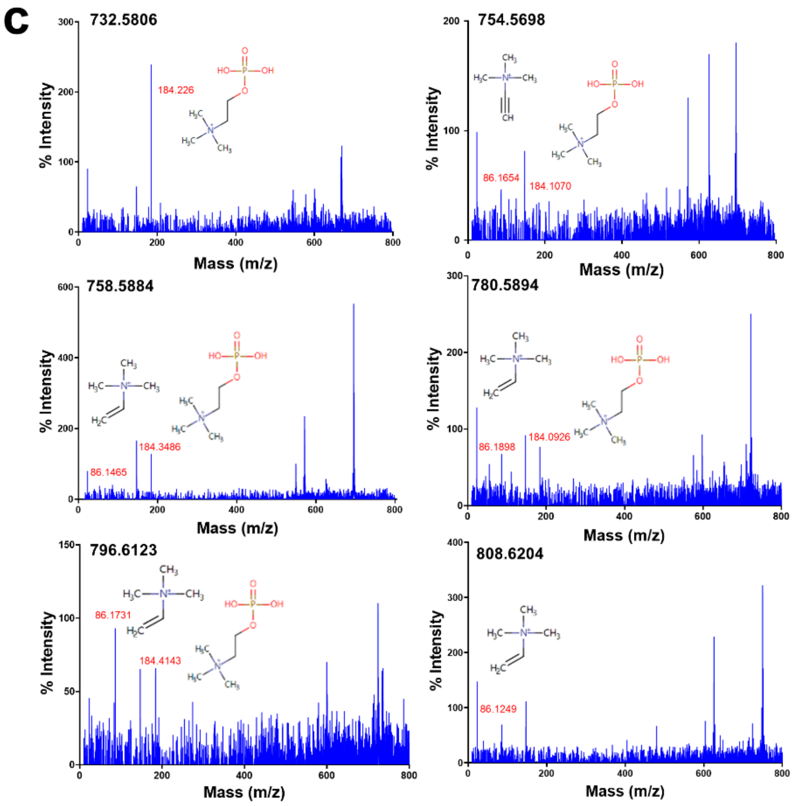

Supplementary Fig. S6 | Phospholipid identification

a, Mass spectrum of sample and buffer. The distinct mass spectrum are indicated.

b, List of identified PC molecules with different molecular weight. The data are searched from HMDB.

c, A further identification of each distinct items in (a). The characteristic groups of PC mass spectrum are based on HMDB.

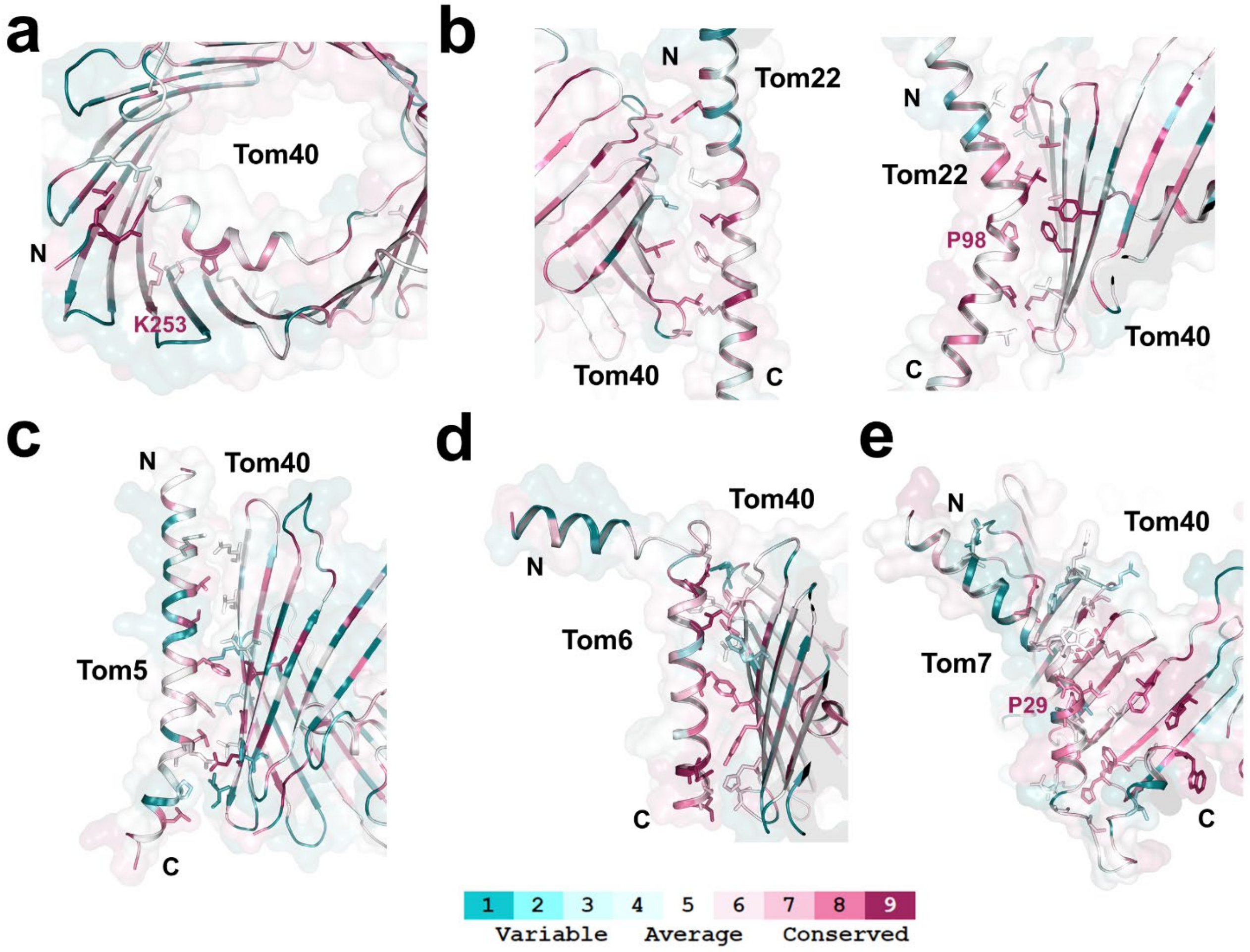

Supplementary Fig. S7 | Surface conservation analyses of Tom subunits.

a, Surface conservation analyses of the interface between Tom40$^{L77-K102}$ and β-strand domains of Tom40. The conserved residue K253 is indicated. Surface representation with residues coloured based on conservation level. The surface conservation analyses are generated by ConSurf, the same below. Shown in IMS view.

b, Surface conservation analyses of the interfaces between Tom40 and Tom22 in both sides. The conserved residue P98 is indicated in the right panel. Shown in side views.

c, Surface conservation analyses of the interface between Tom40 and Tom5. Shown in side view.

d, Surface conservation analyses of the interface between Tom40 and Tom6. Shown in side view.

e, Surface conservation analyses of the interface between Tom40 and Tom7. The conserved residue P29 is indicated. Shown in side view.

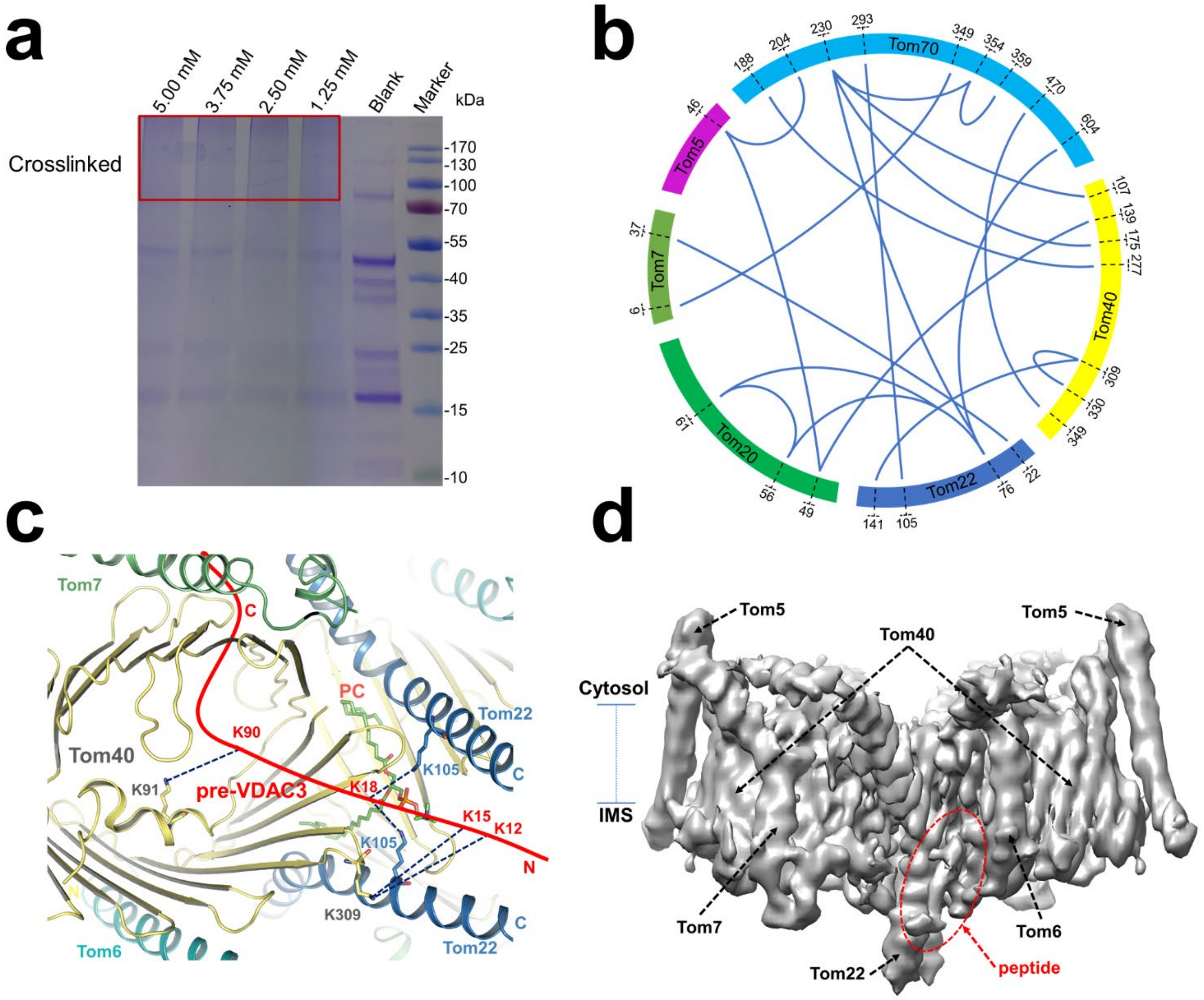

Supplementary Fig. S8 | CL-MS analyses and an unusual map between Tom22 and Tom6.

a, The SDS-PAGE of cross-linked TOM complex. The final crosslinker concentration is 1.25 mM, 2.50 mM, 3.75 mM, and 5 mM, respectively. The samples for detection are indicated with dotted box in red.

b, Interactions between Tom subunits detected by CL-MS. The crosslinked sites are indicated.

c, Diagram for a potential transport pathway of pre-VDAC3 based on CL-MS analyses. The crosslinked sites are indicated.

d, The potential short peptide is indicated with dotted cycles. Tom subunits are indicated. Shown in side view.

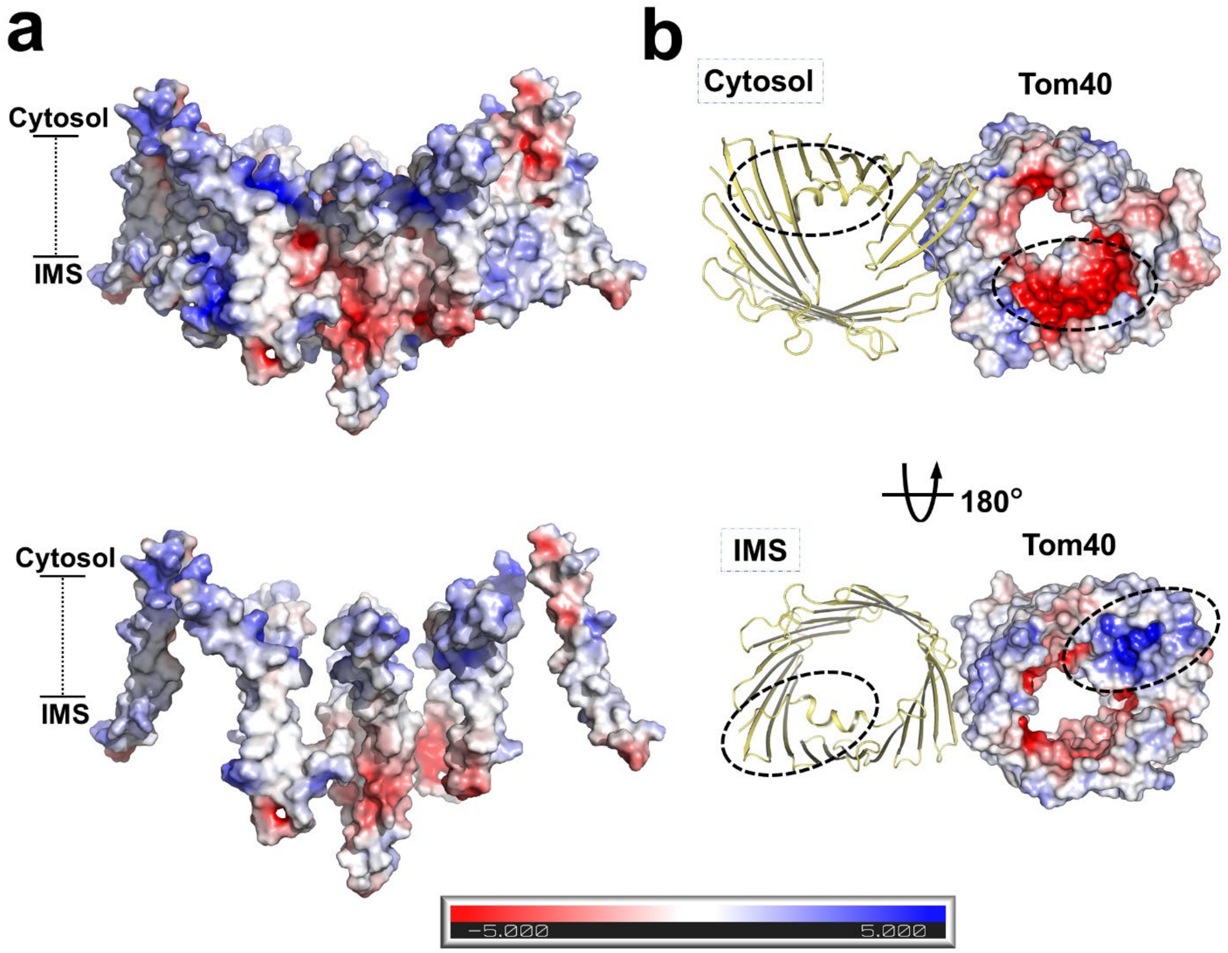

Supplementary Fig. S9 | Surface electrostatics of TOM-CC.

a, Surface electrostatic potential of helical Tom subunits. Side views are shown.

b, Surface electrostatic potential of Tom40. The distinct potential regions are indicated with dotted cycles, corresponding regions of copy units in model are also indicated, respectively. Shown are cutaway side views.

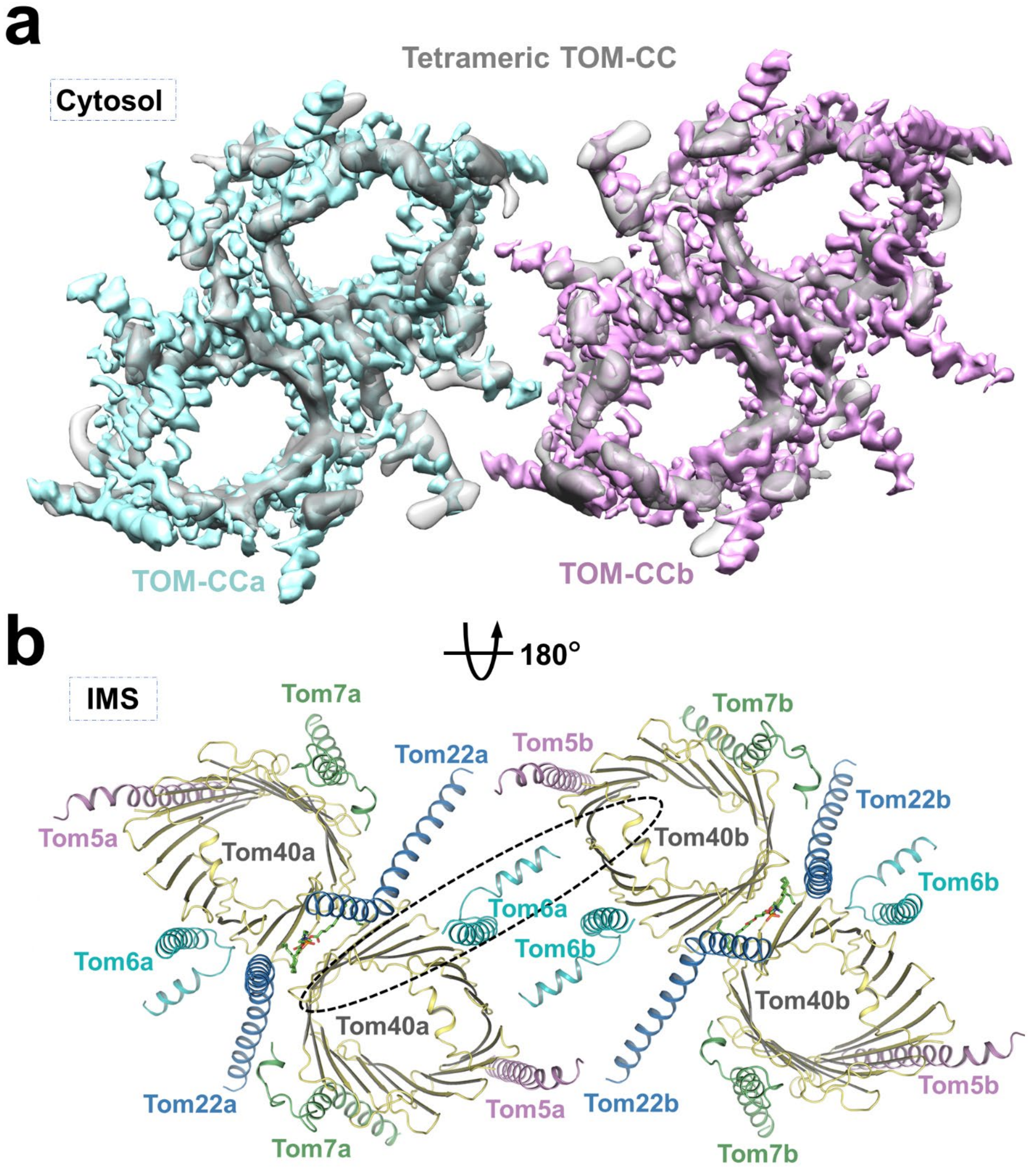

Supplementary Fig. S10 | Tetrameric TOM complex

a, Dimeric TOM complex maps fitted in the tetramer map. Two dimeric units are indicated by a and b. Cytosol views are shown.

b, Fitted model of tetrameric TOM complex. The tetrameric arrangement blurs the boundary between the two proposed exit sites of the TOM complex, which is indicated with dotted cycles. IMS views are shown.

Supplementary Table S1 | Cryo-EM data collection, refinement and validation statistics

**Data collection and processing**		
Microscope	Tian Krios TEM (Thermo Fisher)	
Camera	Gatan K2 Summit direct electron detector	
Energy filter	Gatan GIF Quantum, 20eV slit	
Magnification (calibrated)	130,000 X	
Voltage	300 kV	
Defocus range (μm)	-1.3 - -2.3	
Total electron exposure (e-/Å2)	50	
Exposure rate (e-/pixel/sec)	10	
Number of frames collected	32	
Pixel size (Å)	1.08	
**Reconstruction**		
Software	cryoSPARC and RELION	
Micrographs used	5,443	
Total extracted particles	1,636,577	
Total refined particles	159,369	11,184
Symmetry applied	C2	
Resolution of unmasked/masked reconstructions at 0.5 FSC (Å)	3.68/3.48	12.17/9.59
Resolution of unmasked/masked reconstructions at 0.143 FSC (Å)	3.36/3.43	9.12/8.53
Local resolution range (Å)	2.4-4.0	
**Real space refinement**		
Software	phenix_real_spac_refine in the PHENIX	
Resolution (Å)	3.43	8.53
Model composition		
Number of protein atoms	7,696	15,392
Number of ligand atoms	1	2
CC (box)	0.6	0.48
B factor of protein residues (mean)	102.83	222.80
B factor of protein ligand (mean)	21.3	35.4
R.M.S deviations		
Bond lengths (Å)	0.006	0.006
Bond angles (Å)	0.838	1.047
**Validation**		
Molprobity Score	2.09	3.31
Clashscore	9.19	35.87
Rotamer outliers (%)	1.25	7.75
C-beta deviations	0	0
CaBLAM outliers (%)	5.24	5.24
Ramachandran plot statics		
Favored (%)	90.76	88.09
Allowed (%)	9.14	11.60
Outliers (%)	0.1	0.31

Supplementary Table S2 | CL-MS analyses

Cross-linked peptides of Tom subunits.

Subunits	Cross-linked sequences	Lys-Lys$^{\alpha}$
Tom40-Tom40	E[K]KLPPLPLTL-GYQLDLP[K]ANL	330-309
Tom40-Tom22	GYQLDLP[K]ANLL-PSLPG[K]I	309-141
Tom40-Tom20	VGT[K]QL-A[K]ERAGLSKL	139--49
Tom40-Tom70	H[K]ASDQL-NPKYV[K]AL	277-188
Tom40-Tom70	LNHR[K]NKF-EEVI[K]KF	349-470
Tom40-Tom70	GPGLRS[K]MAIQTQQSKF-AD[K]VLKLL	175-230
Tom40-Tom70	TVN[K]GL-AD[K]VLKLL	107-230
Tom20-Tom5	A[K]ERAGL-[K]KLDSI	49-46
Tom20-Tom20	[K]DAEAVQKF-S[K]LPDL	61-56
Tom22-Tom20	SAAGATFDLSLFVAQ[K]MYR-AGLS[K]LPDLK	76-56
Tom22-Tom20	SAAGATFDLSLFVAQ[K]MYR-LPDL[K]DAEAVQK	76-61
Tom22-Tom22	VAQ[K]MY-VAQ[K]MY	76-76
Tom22-Tom70	VAQ[K]MYRFSRAAL-[K]PPTL	76-604
Tom22-Tom70	PVVFETE[K]L-EV[K]ENSGYL	105-293
Tom22-Tom70	VAQ[K]MYRF-AD[K]VL	76-230
Tom70-Tom70	[K]EANVKLRANAL-D[K]VISL	359-354
Tom70-Tom5	DNK[K]ECL-[K]KLDSI	204-46
Tom70-Tom70	D[K]VISL-AD[K]VL	354-230
Tom70-Tom7	IGNANAA[K]PDL-S[K]EAKQRLQQL	349-6
Tom22-Tom7	P[K]GDAEKPEEEL-[K]RGADPGMPEPTVLSL	22-37

Cross-linked peptides of Tom subunits and VDAC1-3.

Subunits	Cross-linked sequences	Lys-Lys$^{\alpha}$
Tom40-VDAC1	VGT[K]QL-SALLDGKNVNAGGH[K]L	139-274
Tom40-VDAC2	AG[K]YTL-[K]WCEYGL	253-74
Tom40-VDAC3	P[K]ANLLF-G[K]AAKDVF	309-12
Tom40-VDAC3	P[K]ANLLF-GKAA[K]DVF	309-15
Tom40-VDAC3	EECHRKC[K]EL-EN[K]LAEGL	91-90
Tom22-VDAC3	VLPVVFETE[K]L-N[K]GYGFGMVKIDL	105-20
Tom22-VDAC1	PKGDAE[K]PEEEL-ARGL[K]L	27-96
Tom70-VDAC1	VH[K]GL-[K]TKSENGL	516-32
Tom20-VDAC3	QMLLT[K]L-SALIDG[K]NF	125-266

Cross-linked peptides of Tom subunits and Tim subunits.

Subunits	Cross-linked sequences	Lys-Lys$^{\alpha}$
Tom40-Tim44	AG[K]YTLNNWL-TD[K]VTDL	253-282
Tom40-Tim29	P[K]ANLL-QKEK[K]DRLAL	309-242
Tom40-Tim9	DLP[K]ANLL-LDCV[K]DF	309-34
Tom40-Tim14	VGT[K]QL-YRGGFEP[K]MTKREAAL	139-57
Tom40-Tim44	TVN[K]GL-DNV[K]QEL	107-68
Tom40-Tim44	RS[K]MAIQTQQSKF-TD[K]VTDLLGGL	175-282
Tom40-Tim14	AG[K]YTL-SGGYYRGGFEP[K]MTKREAALIL	253-57
Tom70-Tim29	AD[K]VL-[K]PVVL	230-218
Tom22-Tim9	ETE[K]L-AA[K]AGL	105-81
Tom70-Tim50	[K]NREPL-EKQ[K]EVDAL	245-457
Tom40-DNAJC7	IVGATLEK[K]L-I[K]AYL	331-329
Tom70-DNAJC7	D[K]VISL-[K]AECLAML	354-182
Tom70-DNAJC7	[K]QDLDRGLEL-MSLLEE[K]L	524-252
Tom70-Tim29	[K]QDLDRGLEL-Q[K]EKKDRLAL	524-239
Tom70-MIC60	AQAQ[K]CFAL-LKA[K]EEL	441-299
Tom70-Tim50	[K]AKQY-[K]EHCVY	300-265
Tom70-Tim50	NP[K]YVKAL-SD[K]IAEKL	185-247
Tom70-Tim16	LAD[K]VL-MA[K]YL	230-3
Tom70-Tim23	VH[K]GL-Y[K]CTGGL	516-169
Tom70-Tim23	AD[K]VL-EHM[K]GSL	230-201
Tom70-MIC60	D[K]VISL-[K]GMSVSDLADKLSTDDLNSL	354-385
Tom70-MIC60	D[K]VISL-LSVEAL[K]Y	354-577
Tom20-MIC60	S[K]LPDL-[K]PPPEL	56-675
Tom70-SAM50	EQLQ[K]W-F[K]PRPGNF	168-178

Supplementary Table S3 | Model completeness of Tom subunits

Subunit	Mature protein	Chain ID	Modelled residues	Assigned residues
TOM40	361aa	A/B	77-360aa	77-360aa
TOM22	141aa	C/D	49-118aa	56-118aa
TOM7	55aa	G/H	6-57aa	6-54aa
TOM6	73aa	E/F	23-74aa	23-66aa
TOM5	51aa	I/J	12-49aa	12-49aa

Raw Data Gel

Fig. 2f

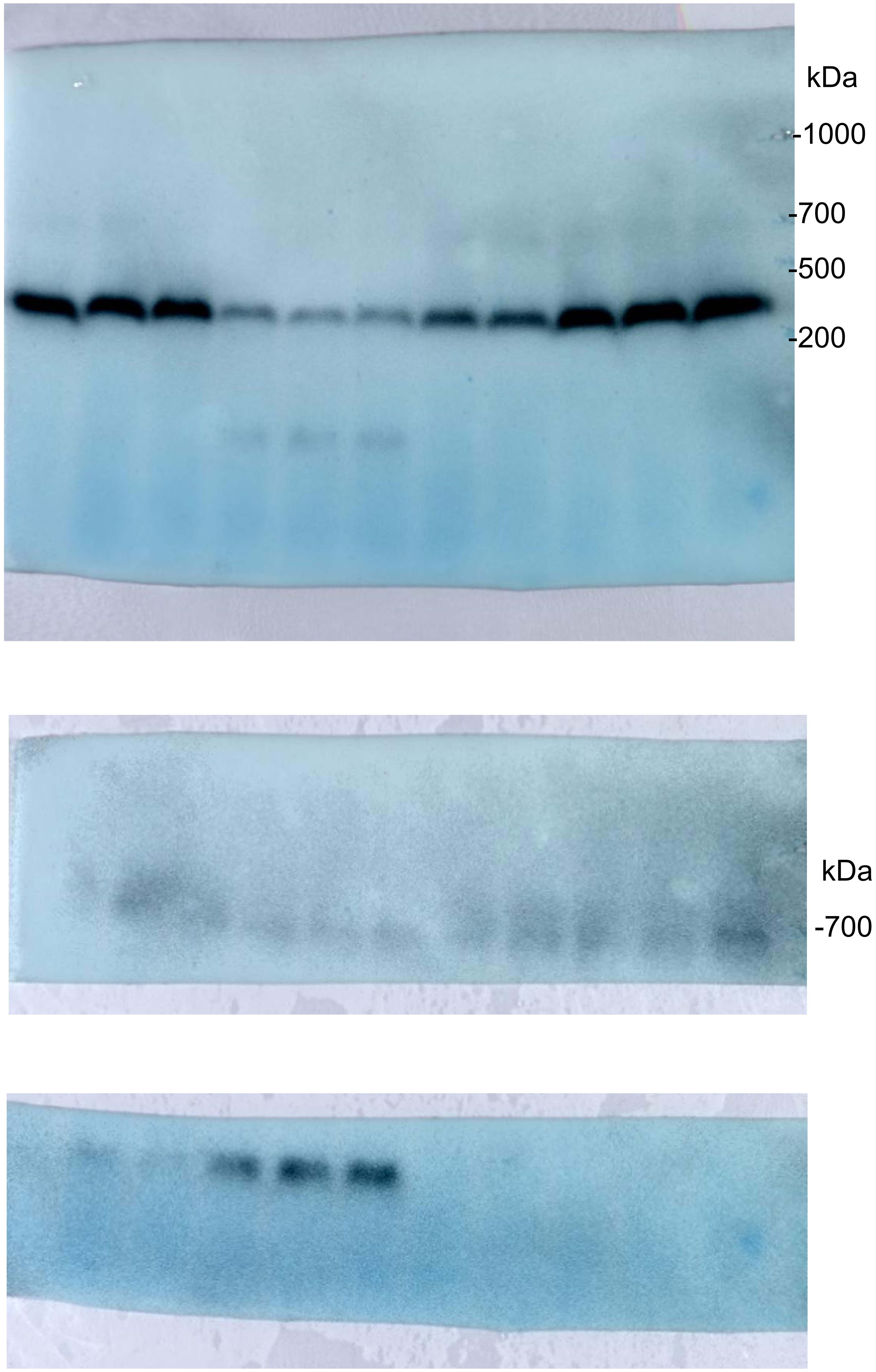

Fig. 2g

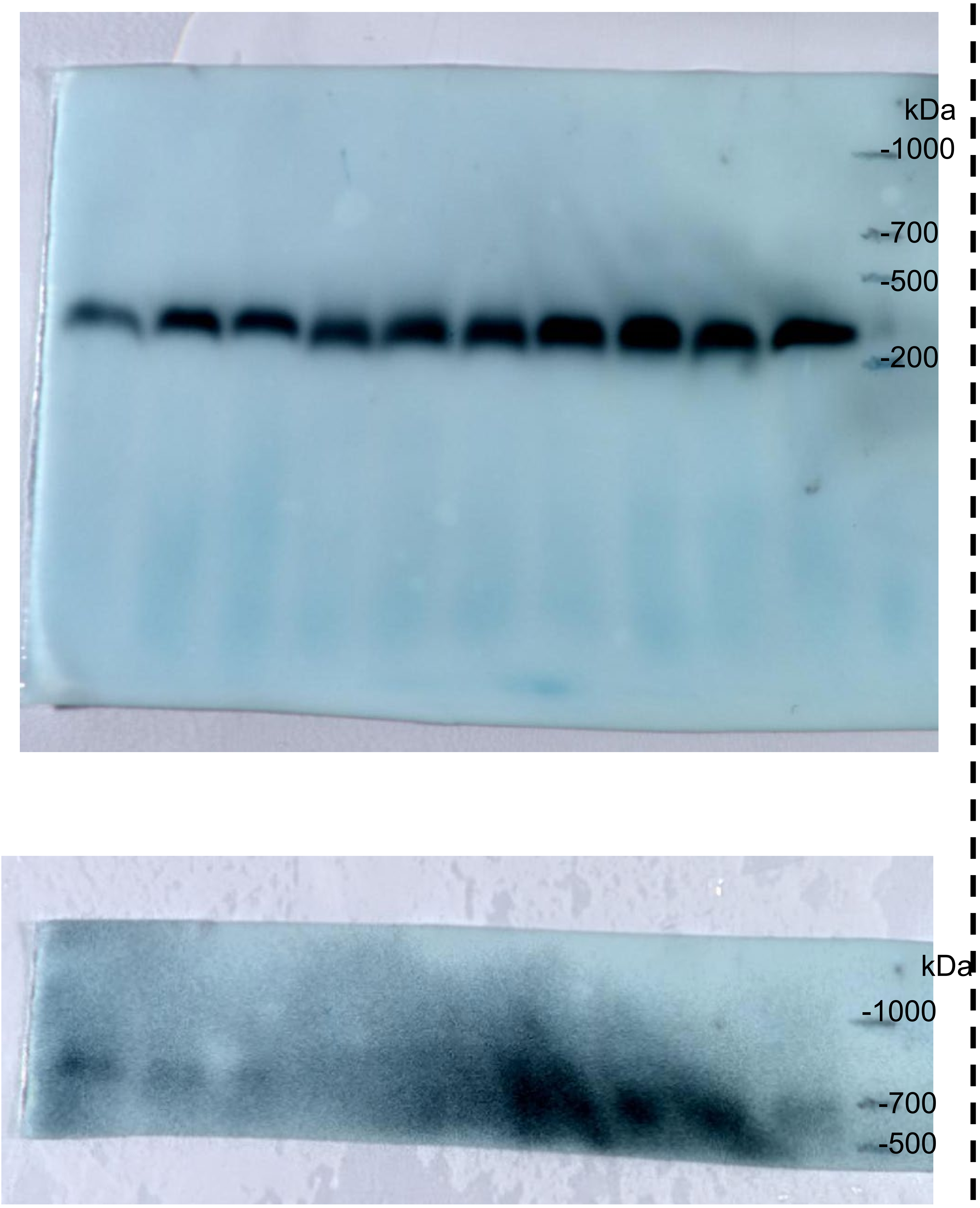

Supplementary Fig. S1b

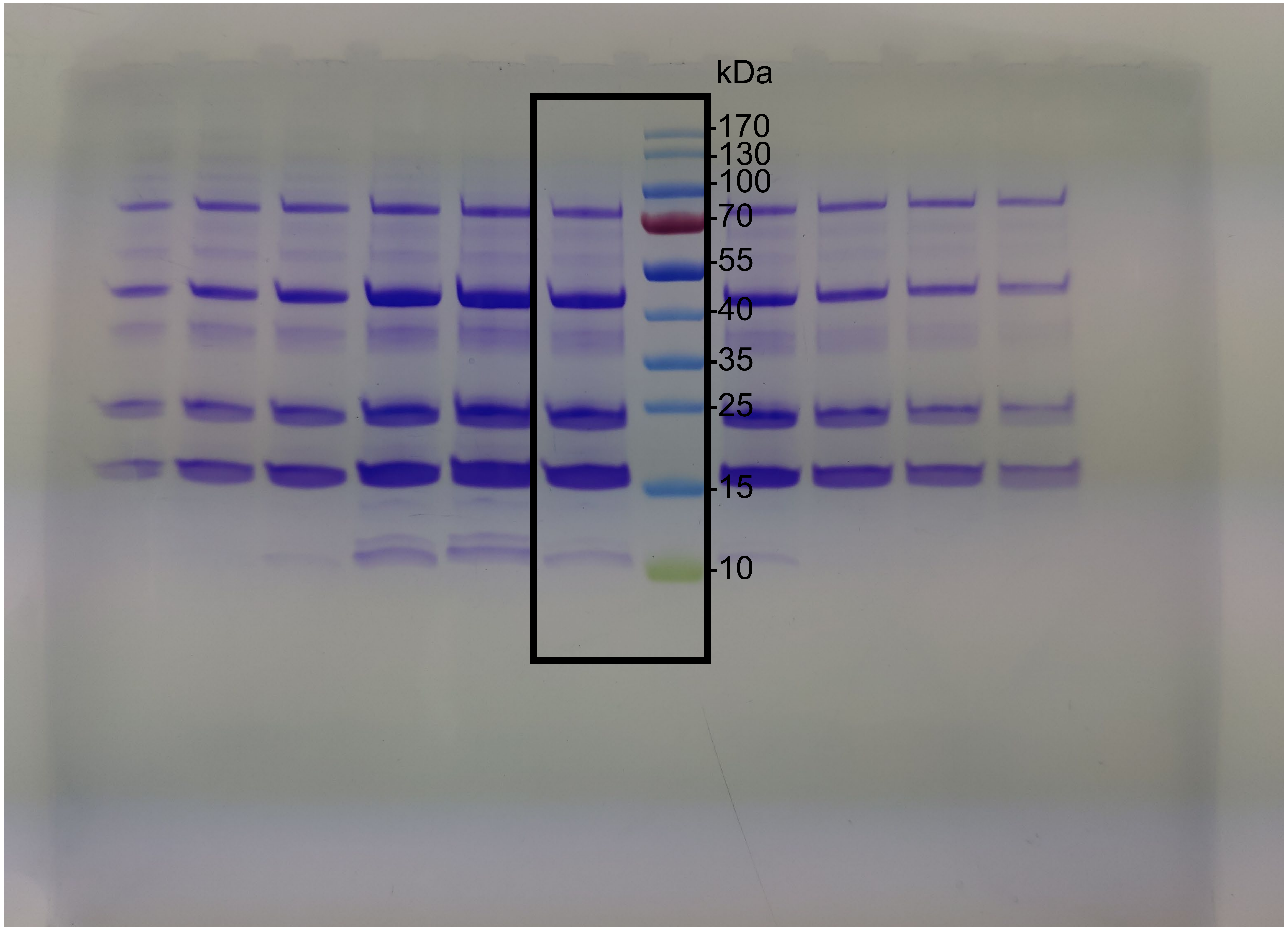

Supplementary Fig. S1c

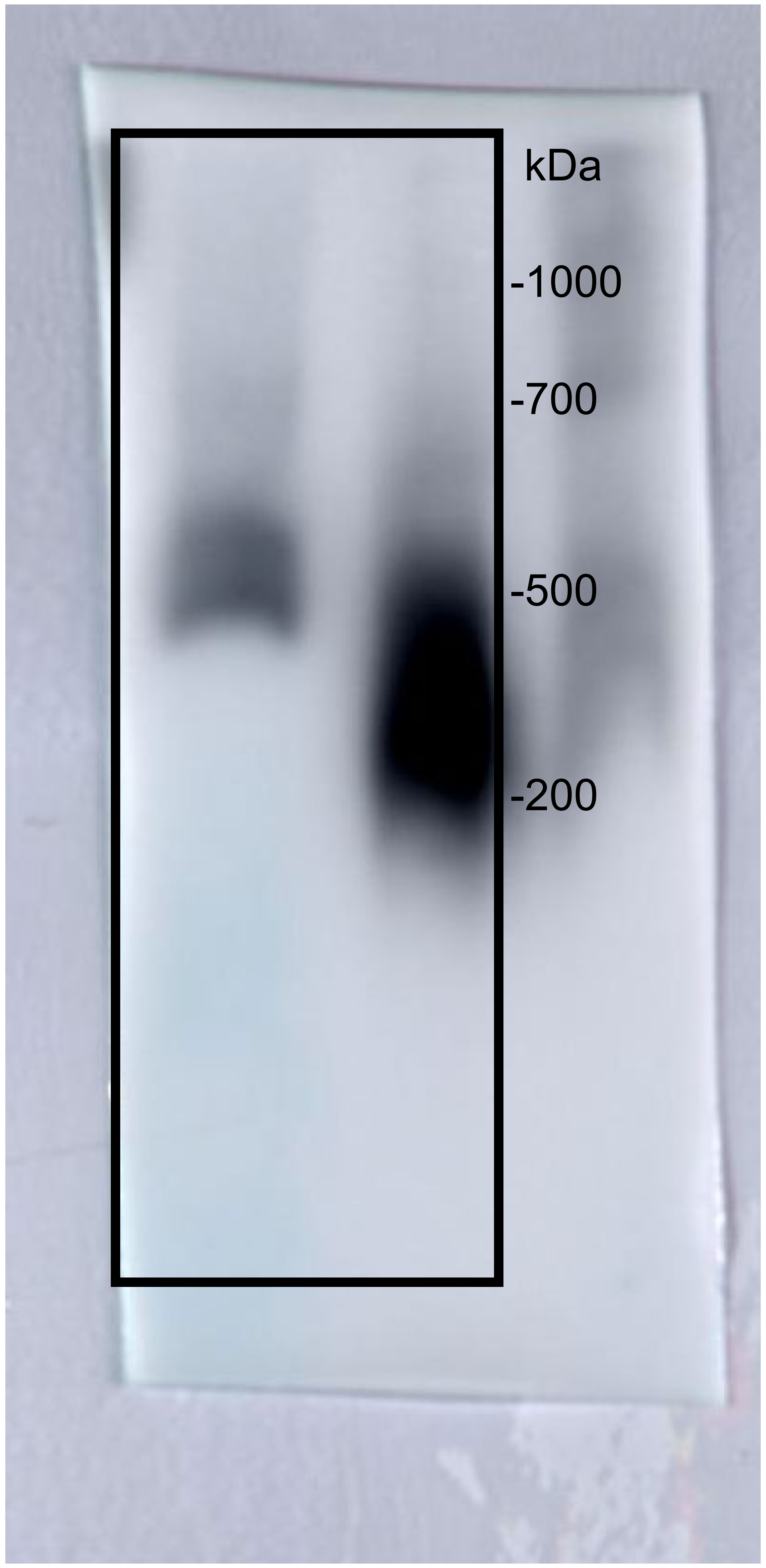

Supplementary Fig. S8a

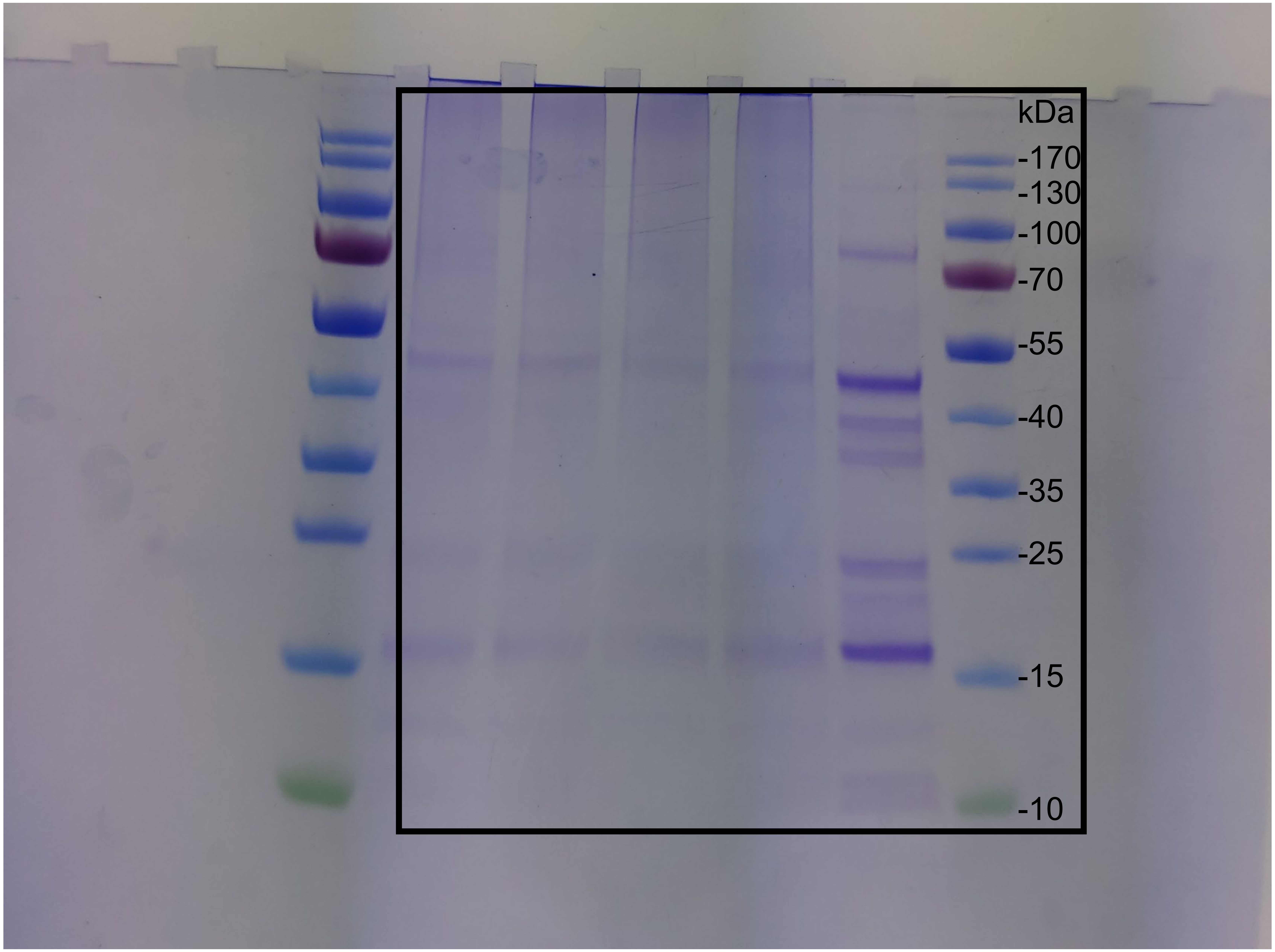

Checked	Confidenc	Max. Xlink	Sequence A	Accession A	Position A	Sequence B	Accession B	Position B
FALSE	High	59.82	A[K]ERAGL	Q15388	49	[K]KLDSI	Q8N4H5	46
FALSE	High	55.39	RS[K]MAIQ	O96008	175	ARGL[K]LTF	P21796	96
FALSE	High	53.17	[K]EANVKLI	O94826	359	D[K]VISL	O94826	354
FALSE	High	50.16	[K]DAEAVC	Q15388	61	S[K]LPDL	Q15388	56
FALSE	High	47.04	GYQLDLP[K	O96008	309	PSLPG[K]I	Q9NS69	141
FALSE	High	40.04	DNK[K]ECL	O94826	204	[K]KLDSI	Q8N4H5	46
FALSE	High	30.72	[K]AECLAM	Q99615	182	D[K]VISL	O94826	354
FALSE	High	30.72	[K]TKSENG	P21796	32	VH[K]GL	O94826	516
FALSE	High	29.35	E[K]KLPPLP	O96008	330	GYQLDLP[K	O96008	309
FALSE	High	29.32	D[K]VISL	O94826	354	AD[K]VL	O94826	230
FALSE	High	28.42	AG[K]YTLN	O96008	253	TD[K]VTDL	O43615	282
FALSE	High	28.09	VAQ[K]MYI	Q9NS69	76	VAQ[K]MY	Q9NS69	76
FALSE	High	27.1	N[K]GYGFG	Q9Y277	20	VLPVVFETE	Q9NS69	105
FALSE	High	26.6	A[K]ERAGL	Q15388	49	VGT[K]QL	O96008	139
FALSE	High	26.6	Q[K]EKKDR	Q9BSF4	239	[K]QDLDRG	O94826	524
FALSE	High	26.6	QKEK[K]DR	Q9BSF4	242	P[K]ANLL	O96008	309
FALSE	High	25.83	AQAQ[K]CF	O94826	441	LKA[K]EEL	Q16891	299
FALSE	High	25.17	DLP[K]ANLI	O96008	309	LDCV[K]DF	Q9Y5J7	34
FALSE	High	25.17	G[K]AAKDV	Q9Y277	12	P[K]ANLLF	O96008	309
FALSE	High	25.17	GKAA[K]DV	Q9Y277	15	P[K]ANLLF	O96008	309
FALSE	High	24.64	VAQ[K]MYI	Q9NS69	76	[K]PPTL	O94826	604
FALSE	High	24.3	SALLDG[K]I	P21796	266	VGT[K]QL	O96008	139
FALSE	High	23.6	[K]RGADPG	Q9P0U1	37	P[K]GDAEK	Q9NS69	22
FALSE	High	23.56	PVVFETE[K	Q9NS69	105	EV[K]ENSG	O94826	293
FALSE	High	23.48	[K]EHCVY	Q02776	265	[K]AKQY	O94826	300
FALSE	High	23.48	IVGATLEK[I	O96008	331	I[K]AYL	Q99615	329
FALSE	High	22.86	EKQ[K]EVD	Q02776	457	[K]NREPL	O94826	245
FALSE	High	22.86	SALLDGKN	P21796	274	VGT[K]QL	O96008	139
FALSE	High	22.86	LAD[K]VL	O94826	230	MA[K]YL	Q9Y3D7	3
FALSE	High	22.86	LQLQW[K](	O94826	524	AEGL[K]L	Q9Y277	96
FALSE	High	22.86	YRGGFEP[K	Q96DA6	57	VGT[K]QL	O96008	139
FALSE	High	22.1	Y[K]CTGGL	O14925	169	VH[K]GL	O94826	516
FALSE	High	22.1	NPKYV[K]A	O94826	188	H[K]ASDQL	O96008	277
FALSE	High	21.53	S[K]EAKQR	Q9P0U1	6	IGNANAA[K	O94826	349
FALSE	High	21.4	[K]WCEYGL	P45880	74	AG[K]YTL	O96008	253
FALSE	High	21.4	DNV[K]QEL	O43615	68	TVN[K]GL	O96008	107
FALSE	High	21.4	SALIDG[K]N	Q9Y277	266	QMLLT[K]L	Q15388	125
FALSE	High	21.4	EHM[K]GSL	O14925	201	AD[K]VL	O94826	230
FALSE	High	21.4	LNHR[K]NK	O96008	349	EEVI[K]KF	O94826	470
FALSE	High	21.23	[K]GMSVSD	Q16891	385	D[K]VISL	O94826	354
FALSE	High	20.9	RS[K]MAIQ	O96008	175	TD[K]VTDLI	O43615	282
FALSE	High	20.81	PKGDAE[K]	Q9NS69	27	ARGL[K]L	P21796	96
FALSE	High	20.81	[K]PPPEL	Q16891	675	S[K]LPDL	Q15388	56
FALSE	High	20.81	F[K]PRPGN	Q9Y512	178	EQLQ[K]W	O94826	168
FALSE	High	20.79	[K]PVVL	Q9BSF4	218	AD[K]VL	O94826	230
FALSE	High	20.78	AA[K]AGL	Q9Y5J7	81	ETE[K]L	Q9NS69	105
FALSE	High	20.38	SGGYYRGG	Q96DA6	57	AG[K]YTL	O96008	253
FALSE	High	20.28	S[K]EAKQR	Q9P0U1	6	VGT[K]QL	O96008	139
FALSE	High	20.28	VAQ[K]MYI	Q9NS69	76	AD[K]VL	O94826	230

FALSE	High	20.28	MPSPQFI[K	O94826	258	L[K]NAQLE	Q99615	368
FALSE	High	20.16	GPGLRS[K]	O96008	175	AD[K]VLKLI	O94826	230
FALSE	High	20.16	EECHRKC[K	O96008	91	EN[K]LAEG	Q9Y277	90
FALSE	High	20.16	AD[K]VLKLI	O94826	230	TVN[K]GL	O96008	107
FALSE	High	20.16	SD[K]IAEKL	Q02776	247	NP[K]YVKA	O94826	185

Crosslinker	Crosslink T	# CSMs	Protein Descriptions A	Protein Descriptions B
DSS	Inter	1	Q15388	Q8N4H5
DSS	Inter	7	O96008	P21796
DSS	Intra	4	O94826	O94826
DSS	Intra	6	Q15388	Q15388
DSS	Inter	6	O96008	Q9NS69
DSS	Inter	10	O94826	Q8N4H5
DSS	Inter	9	Q99615	O94826
DSS	Inter	2	P21796	O94826
DSS	Intra	1	O96008	O96008
DSS	Intra	1	O94826	O94826
DSS	Inter	1	O96008	O43615
DSS	Intra	1	Q9NS69	Q9NS69
DSS	Inter	1	Q9Y277	Q9NS69
DSS	Inter	2	Q15388	O96008
DSS	Inter	1	Q9BSF4	O94826
DSS	Inter	1	Q9BSF4	O96008
DSS	Inter	1	O94826	Q16891
DSS	Inter	4	O96008	Q9Y5J7
DSS	Inter	1	Q9Y277	O96008
DSS	Inter	1	Q9Y277	O96008
DSS	Inter	1	Q9NS69	O94826
DSS	Inter	2	P21796	O96008
DSS	Inter	1	Q9P0U1	Q9NS69
DSS	Inter	1	Q9NS69	O94826
DSS	Inter	1	Q02776	O94826
DSS	Inter	1	O96008	Q99615
DSS	Inter	1	Q02776	O94826
DSS	Inter	1	P21796	O96008
DSS	Inter	1	O94826	Q9Y3D7
DSS	Inter	1	O94826	Q9Y277
DSS	Inter	1	Q96DA6	O96008
DSS	Inter	1	O14925	O94826
DSS	Inter	1	O94826	O96008
DSS	Inter	1	Q9P0U1	O94826
DSS	Inter	2	P45880	O96008
DSS	Inter	1	O43615	O96008
DSS	Inter	1	Q9Y277	Q15388
DSS	Inter	1	O14925	O94826
DSS	Inter	1	O96008	O94826
DSS	Inter	1	Q16891	O94826
DSS	Inter	1	O96008	O43615
DSS	Inter	1	Q9NS69	P21796
DSS	Inter	1	Q16891	Q15388
DSS	Inter	1	Q9Y512	O94826
DSS	Inter	1	Q9BSF4	O94826
DSS	Inter	1	Q9Y5J7	Q9NS69
DSS	Inter	1	Q96DA6	O96008
DSS	Inter	1	Q9P0U1	O96008
DSS	Inter	1	Q9NS69	O94826

DSS	Inter	1	O94826	Q99615
DSS	Inter	1	O96008	O94826
DSS	Inter	1	O96008	Q9Y277
DSS	Inter	1	O94826	O96008
DSS	Inter	1	Q02776	O94826

Dimeric TOM complex BN-PAGE Mass Spectrum

Accession	Description	Score
O96008	Mitochondrial import receptor subunit TOM40 homolog OS=Homo sapiens GN	17953.35
Q9NS69	Mitochondrial import receptor subunit TOM22 homolog OS=Homo sapiens GN	5711.02
Q8N4H5	Mitochondrial import receptor subunit TOM5 homolog OS=Homo sapiens GN=	1146.37
Q9P0U1	Mitochondrial import receptor subunit TOM7 homolog OS=Homo sapiens GN=	466.47
Q15388	Mitochondrial import receptor subunit TOM20 homolog OS=Homo sapiens GN	463.45
Q96B49	Mitochondrial import receptor subunit TOM6 homolog OS=Homo sapiens GN=	213.82

Coverage	# Proteins	# Unique Peptides	# Peptides	# PSMs	# AAs
85.60	2	21	38	3690	361
100.00	2	12	12	1388	142
100.00	4	16	16	802	51
83.64	3	2	10	246	55
86.90	1	12	12	114	145
94.59	1	6	6	83	74

MW [kDa]	calc. pI
37.9	7.25
15.5	4.34
6.0	9.70
6.2	10.29
16.3	8.60
8.0	4.89

SDS-PAGE Mass Spectrum

Accession	Description	Score	Coverage	# Proteins
O94826	Mitochondrial import receptor subunit TOM70 OS=Homo sapiens GN=TOM	1695.56	78.29	1
O96008	Mitochondrial import receptor subunit TOM40 homolog OS=Homo sapiens	6138.80	73.41	3
Q9NS69	Mitochondrial import receptor subunit TOM22 homolog OS=Homo sapiens	1946.82	97.89	1
Q15388	Mitochondrial import receptor subunit TOM20 homolog OS=Homo sapiens	987.83	87.59	1
Q8N4H5	Mitochondrial import receptor subunit TOM5 homolog OS=Homo sapiens	181.55	64.71	4
Q9P0U1	Mitochondrial import receptor subunit TOM7 homolog OS=Homo sapiens	34.50	70.91	2
Q96B49	Mitochondrial import receptor subunit TOM6 homolog OS=Homo sapiens	29.17	32.43	1

Unique Peptid	# Peptides	# PSMs	# AAs	MW [kDa]	calc. pI
55	55	707	608	67.4	7.12
30	30	1686	361	37.9	7.25
13	13	736	142	15.5	4.34
16	16	396	145	16.3	8.60
8	8	95	51	6.0	9.70
2	7	20	55	6.2	10.29
3	3	14	74	8.0	4.89

Tetrameric TOM complex BN-PAGE Mass Spectrum

Accession	Description	Score	Coverage
O96008	Mitochondrial import receptor subunit TOM40 homolog OS=Homo s	1680.83	79.78
Q9NS69	Mitochondrial import receptor subunit TOM22 homolog OS=Homo s	766.28	78.17
Q15388	Mitochondrial import receptor subunit TOM20 homolog OS=Homo s	83.97	40.69
Q8N4H5	Mitochondrial import receptor subunit TOM5 homolog OS=Homo sa	72.53	86.27
Q96B49	Mitochondrial import receptor subunit TOM6 homolog OS=Homo sa	25.78	32.43
Q9P0U1	Mitochondrial import receptor subunit TOM7 homolog OS=Homo sa	5.00	43.64

# Proteins	# Unique Peptides	# Peptides	# PSMs	# AAs	MW [kDa]
3	22	22	522	361	37.9
2	7	7	285	142	15.5
1	5	5	38	145	16.3
4	9	9	53	51	6.0
1	3	3	16	74	8.0
4	3	3	5	55	6.2

calc. pI
7.25
4.34
8.60
9.70
4.89
10.29
